# An Integrated Metabolomic and Gene Expression Analysis of ‘Sachinoka’ Strawberry and Its Somaclonal Mutant Reveals Fruit Color and Volatiles Differences

**DOI:** 10.3390/plants12010082

**Published:** 2022-12-23

**Authors:** Ruiqing Bian, Shuang Yu, Xinyu Song, Jinxiang Yao, Junxiang Zhang, Zhihong Zhang

**Affiliations:** 1 Liaoning Key Laboratory of Strawberry Breeding and Cultivation, College of Horticulture, Shenyang Agricultural University, Shenyang 110866, China; 2 Laboratory of Protected Horticulture (Shenyang Agricultural University), Ministry of Education, Shenyang 110866, China; 3 Analytical and Testing Center, Shenyang Agricultural University, Shenyang 110866, China

**Keywords:** strawberry, aroma, somaclonal variation, volatile components, white flesh

## Abstract

Plant tissue culture produces a wide range of genetic variations which are useful for quality improvement of the plant species. However, the differences in metabolic components and the key genes responsible for the difference in metabolic components between somaclonal variation and the original parent are still largely unknown. In this study, a mutant named ‘Mixue’ was identified with somaclonal variation of the ‘Sachinoka’ strawberry. The contents of pelargonidin-3-*O*-glucoside and cyanidin-3-*O*-glucoside in the red fruit of ‘Mixue’ were significantly decreased compared with ‘Sachinoka’. In comparison with ‘Sachinoka’, the expression levels of *FaMYB10*, *FaMYB11.2*, *FaWD40* and *FaTT19* in the turning fruit of ‘Mixue’ were significantly down-regulated, while the expression of *FaMYB1* was significantly up-regulated in the red fruit. ‘Sachinoka’ and ‘Mixue’ fruits were found to have 110 volatile components. Among them, 15 volatile components in the red fruit of ‘Mixue’ were significantly increased compared with ‘Sachinoka’, such as nerolidol, benzaldehyde, ethyl hexanoate, ethyl isovalerate, which led to an enhanced aroma in ‘Mixue’ and might result from the up-regulated expression of *FaNES1*, *FaCNL* and *FaAATs* in ‘Mixue’. These results provide useful information on the effect of somaclonal variation on metabolic components of strawberry fruit and lay the foundation for the improvement in quality of strawberry.

## 1. Introduction

Strawberry (*Fragaria × ananassa* Duch.) belongs to the *Fragaria* genus of the Rosaceae family, which is an important economic crop and is widely consumed throughout the world. Antioxidants, vitamins, dietary fibers, and pleasant volatile components are abundant in strawberry. Strawberry also has the potential benefits to human health, such as protective effects in inflammation, neurodegenerative diseases and coronary heart diseases, ageing, cancer, and obesity [1].

Conventional propagation of strawberry is through runner and this kind of propagation produces new clonal plants, which are susceptible to plant diseases caused by various fungi and viruses [2]. In vitro propagation techniques using different explants have been widely applied for efficient strawberry production [3]. However, the propagation of strawberry through tissue culture may produce genetic variations compared with plants obtained through runners [4]. Tissue culture germinating variation in regenerated plants is called somaclonal variation [5]. The occurrence of somaclonal variation is related to point mutations, transposable elements, DNA methylation, changed sequence copy numbers and chromosomal rearrangements [6,7,8]. Somaclonal variation may be affected by explant types, culture media and tissue culture conditions [9]. In addition, plant growth regulators, such as thidiazuron (TDZ), 2,4-dichlorophenoxyacetic acid (2,4-D) or 3-indolebutyric acid (IBA), have been implicated in the induction of somaclonal variability [10,11]. Together, the molecular basis of somaclonal variation is complex and remains uncertain. Interestingly, the molecular mechanism of ‘bud sports’ in woody crop species, also known as somatic mutations, has made some progress [12]. For instance, somatic mutations in the color of grape skin [13], fruit acidity of sweet orange [14], and fruit skin of apple [12] are associated with retrotransposon insertion, DNA transposon insertion and DNA methylation in key factors involved in color and acidity, which in turn regulates the expression of these key factors to affect corresponding phenotypes. The potential of somaclonal variation has yet to be completely exploited for plant improvement by breeders [7]. In addition, the variations in metabolic components between somaclonal variation and the original parent and the key genes responsible for the variations are still largely unknown.

In this study, ‘Sachinoka’ and its somaclonal mutant ‘Mixue’ were used as the research materials. ‘Mixue’ is a rare strawberry somaclonal mutant with white-flesh and enhanced aroma which comes from the tissue culture of apices of runner tips of ‘Sachinoka’. A series of metableolic components and gene expression analysis were conducted to explore the reasons for the differences in flesh color and volatiles between ‘Sachinoka’ and its somaclonal mutant ‘Mixue’. These data will be helpful to understand the effect of somaclonal variation on metableolic components of strawberry fruit and will lay the foundation for quality improvement of strawberry.

## 2. Results

### 2.1. Morphological Characteristics of ‘Sachinoka’ Strawberry and Its Somaclonal Mutant ‘Mixue’

**Figure 1:**
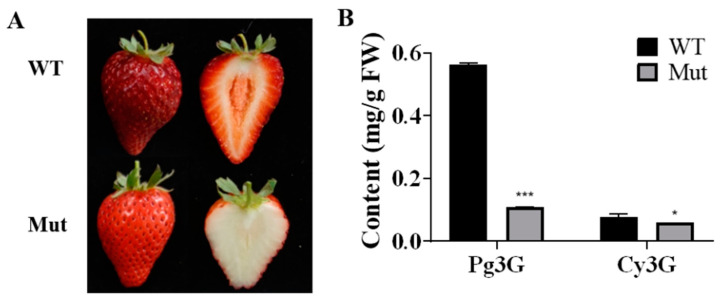
Phenotype (**A**) and anthocyanins content (**B**) analysis of ‘Sachinoka’ strawberry (WT) and its somaclonal mutant ‘Mixue’ (*Mut*) at red fruit stage. * (*p* < 0.05) and *** (*p* < 0.001) represent significant differences between ‘Sachinoka’ strawberry and its somaclonal mutant ‘Mixue’.

The fruit skin and flesh color of ‘Sachinoka’ and its somaclonal mutant ‘Mixue’ were not significantly different at the green and white fruit stages, respectively. During the turning stage, the fruit skin and flesh color of ‘Sachinoka’ were red and light red, respectively, while the fruit skin and flesh color of the somaclonal mutant ‘Mixue’ were light red and white, respectively (Figure S1). At the red fruit stage, the fruit skin and flesh color of ‘Sachinoka’ were dark red and red, respectively, while the fruit skin and flesh color of the somaclonal mutant ‘Mixue’ were red and white, respectively (Figure 1A). The contents of pelargonidin-3-*O*-glucoside (Pg3G) and cyanidin-3-*O*-glucoside (Cy3G) were remarkably increased in the red fruit of ‘Sachinoka’ compared to its somaclonal mutant ‘Mixue’ (Figure 1B). The contents of Pg3G and Cy3G in ‘Sachinoka’ were 5.15 and 1.32 times higher than in its somaclonal mutant ‘Mixue’, which were consistent with more pigment accumulation in ‘Sachinoka’ compared with its somaclonal mutant ‘Mixue’.

### 2.2. The Amount and Content Analysis of Volatile Components in ‘Sachinoka’ Strawberry and Its Somaclonal Mutant ‘Mixue’

To get a chemical insight into the differences in volatile components between ‘Sachinoka’ and its somaclonal mutant ‘Mixue’, volatile components were analyzed in the four developmental stages of ‘Sachinoka’ and its somaclonal mutant ‘Mixue’. A total of 110 volatile components were identified in the four stages of ‘Sachinoka’ and its somaclonal mutant ‘Mixue’, including alcohols (17), acids (9), ethyl esters (10), acetate esters (8), other esters (8), benzene and volatile phenols (15), aldehydes and ketones (23), isoprenoids (17) and furans (3) (Table S3). Some 70, 80, 88, 105 volatile components in ‘Sachinoka’ and its somaclonal mutant ‘Mixue’ were identified in the green, white, turning and red fruits, respectively. These data indicated that the number of volatile components gradually increased and the aroma became more intense during strawberry ripening.

In the red fruit, 15 volatile components were significantly increased in somaclonal mutant ‘Mixue’ with at least 1.5-fold change relative to ‘Sachinoka’, including two alcohols, three ethyl esters, one acetate, on other ester, five aldehydes and ketones, and three isoprenoids (Table S3). Interestingly, the contents of ethyl hexanoate and nonanal with low olfactory threshold in somaclonal mutant ‘Mixue’ were 1.70 and 3.77 times higher than those in ‘Sachinoka’, respectively, which may have some influence on the flavor of ripening strawberry fruit.

### 2.3. The Volatile Components and Trends of ‘Sachinoka’ Strawberry and Its Somaclonal Mutant ‘Mixue’ Fruits at Different Developmental Stages

We analyzed the content of nine categories of volatile components in the fruits of ‘Sachinoka’ and its somaclonal mutant ‘Mixue’ at different developmental stages. In the green fruit stage, the contents of aldehydes and ketones in the somaclonal mutant ‘Mixue’ fruits were significantly higher than those in ‘Sachinoka’. The total contents of 2-hexenal and hexanal in ‘Sachinoka’ and its somaclonal mutant ‘Mixue’ at the green fruit stage accounted for 88.66% and 94.59% of the aldehydes and ketones, respectively. The total contents of 2-hexenal and hexanal in the somaclonal mutant ‘Mixue’ were 4.37 times higher than those in ‘Sachinoka’. In the white fruit stage, the content of acetate ester was increased by 84.79% in ‘Sachinoka’ compared with the green fruit stage and was markedly higher than that in somaclonal mutant ‘Mixue’ (Table S3). In addition, the contents of aldehydes and ketones in the somaclonal mutant ‘Mixue’ were decreased by 65.54% in the white fruit stage compared with the green fruit stage and there was no great difference between ‘Sachinoka’ and its somaclonal mutant ‘Mixue’. During the turning stage, the contents of other esters in the somaclonal mutant ‘Mixue’ were significantly higher than those in ‘Sachinoka’, which were 10.33 times higher than those in ‘Sachinoka’. Among other esters, methyl hexanoate largely accumulated in the somaclonal mutant ‘Mixue’ during the turning stage, which was 21.62 times higher than that in ‘Sachinoka’. During the red stage, the contents of ethyl esters in the somaclonal mutant ‘Mixue’ fruit was significantly higher than that in ‘Sachinoka’. The content of ethyl hexanoate increased 1.70 times in the somaclonal mutant ‘Mixue’ compared with ‘Sachinoka’ (Table S3).

**Figure 2:**
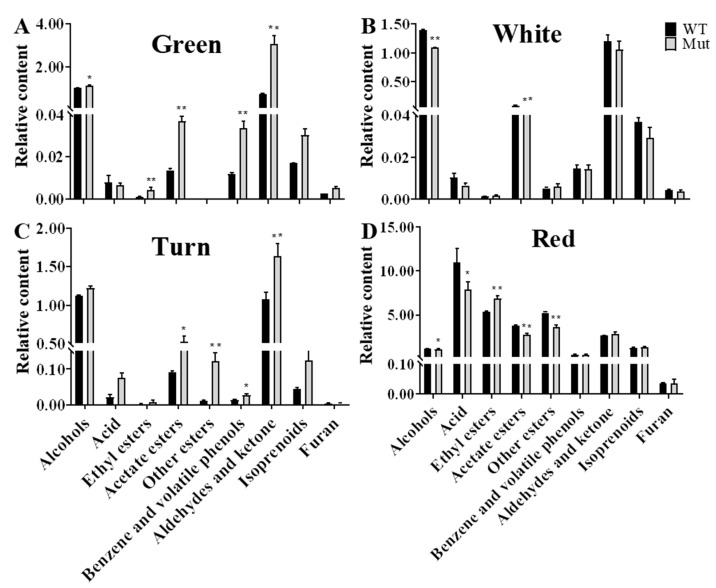
Comparative analysis of the contents of nine categories of volatile components in ‘Sachinoka’ strawberry and its somaclonal mutant ‘Mixue’ at different developmental stages. WT represents cultivated strawberry ‘Sachinoka’. Mut represents somaclonal mutant ‘Mixue’. * (*p* < 0.05) and ** (*p* < 0.01) represent significant differences in volatile components contents between the nine major categories of ‘Sachinoka’ strawberry and its somaclonal mutant ‘Mixue’.

Next, we analyzed the trend of the contents of the nine categories of volatile components in the fruit of the ‘Sachinoka’ and its somaclonal mutant ‘Mixue’ at four developmental stages. In the first three stages of fruit development (green stage, white stage, and turning stage), the trend of the contents of nine categories of volatile components between ‘Sachinoka’ and its somaclonal mutant ‘Mixue’ was similar (Figure 2). The contents of alcohols, aldehydes and ketones accounted for approximately 90% of the total contents of volatile components in the first three stages of fruit development. The contents of acids, ethyl esters and other esters were significantly increased and the contents of alcohols, aldehydes and ketones were significantly decreased in the red stage of ‘Sachinoka’ and its somaclonal mutant ‘Mixue’ fruits compared with the first three stages of fruit development. Acids, other esters, and ethyl esters represented the most abundant components in the red fruit of ‘Sachinoka’ and accounted for 38.78%, 20.87% and 17.01% of the total contents of volatile components, respectively. The top three volatile components with higher contents in the somaclonal mutant ‘Mixue’ fruit during the red stage were acids, ethyl esters and other esters, accounting for 33.31%, 25.98% and 17.14% of the total contents of volatile components, respectively (Table S4).

### 2.4. The Odor Activity Values (OAVs) of the Major Volatile Components in ‘Sachinoka’ Strawberry and Its Somaclonal Mutant ‘Mixue’

Volatile components in strawberry fruit are usually distinguished by the odor activity value [15] (OAV: ratio of concentration to its sensory threshold). Volatile components having OAVs greater than 1 are considered as major contributors to the aroma [16]. Higher OAVs of volatile components indicate a greater contribution to the aroma of strawberry fruit. Based on the published threshold value of aroma substances [17,18,19,20,21], we analyzed the OAVs of 29 volatile components in ‘Sachinoka’ and its somaclonal mutant ‘Mixue’ (Table 1). There were 13 volatile components with OAVs greater than 1 at the red fruit stage of ‘Sachinoka’. In the somaclonal mutant ‘Mixue’, there were 14 volatile components with OAVs greater than 1 during the red fruit stage.

The characteristic volatile components with higher OAVs in ‘Sachinoka’ and its somaclonal mutant ‘Mixue’ during the red fruit stage were ethyl hexanoate, ethyl butyrate and linalool (Table 1). The OAVs of ethyl hexanoate, ethyl isovalerate, nonanal and nerolidol in the red fruit of the somaclonal mutant ‘Mixue’ were 1.70, 2.22, 3.77 and 1.69 times higher compared with ‘Sachinoka’, respectively. The OAV of methyl hexanoate in the red fruit of the somaclonal mutant ‘Mixue’ was lower than that in ‘Sachinoka’ and decreased by 38.55% compared with ‘Sachinoka’. There was no significant difference in the OAVs of linalool in the red fruit of ‘Sachinoka’ and its somaclonal mutant ‘Mixue’. In addition, the OAV of benzaldehyde was greater than 1 in the red fruit stage of somaclonal mutant ‘Mixue’ and less than 1 in the red fruit stage of ‘Sachinoka’ (Table 1).

### 2.5. Unique Volatile Components in the Somaclonal Mutant ‘Mixue’

To further compare the differences in volatile components between ‘Sachinoka’ and its somaclonal mutant ‘Mixue’, a comparative analysis of the unique volatile components in the fruits of ‘Sachinoka’ and its somaclonal mutant ‘Mixue’ at four developmental stages was performed. Nine unique volatile components were identified in ‘Sachinoka’. Among them, seven unique volatile components in the green fruit, one unique volatile component in the white fruit and one unique volatile component in the red fruit were identified, respectively (Table S5). Eleven unique volatile components were identified in the somaclonal mutant ‘Mixue’. Among them, five unique volatile components in the green fruit, two unique volatile components in the white fruit and four unique volatile components in the turning fruit (Table S6). Ethyl butyrate and nerolidol are characteristic volatile components of strawberry. Ethyl butyrate was detected in the green and white fruits of somaclonal mutant ‘Mixue’ fruits with OAVs greater than 1 (Table 1). During the turning stage, nerolidol was only present in the somaclonal mutant ‘Mixue’. In the red stage, nerolidol dramatically accumulated in both ‘Sachinoka’ and its somaclonal mutant ‘Mixue’ fruits. However, the content of nerolidol in the somaclonal mutant ‘Mixue’ was 1.69 times higher than that in ‘Sachinoka’ (Table S3). In addition, ethyl hexanoate is considered as one of the fruit characteristic aroma components in strawberry [22], which was a unique volatile substance in the turning stage of the somaclonal mutant ‘Mixue’ fruit and the OAV was greater than 1 (Table 1). At the red fruit stage, ethyl hexanoate was significantly accumulated in both ‘Sachinoka’ and its somaclonal mutant ‘Mixue’ fruit, while the content of ethyl hexanoate in the somaclonal mutant ‘Mixue’ was 1.70 times higher than that in ‘Sachinoka’ (Table S3).

### 2.6. Principal Component Analysis of Volatile Components

**Figure 3:**
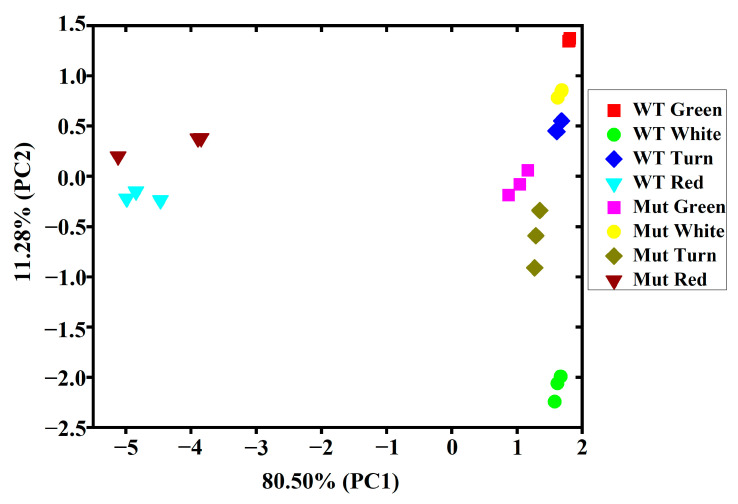
Principal component analysis of the contents of volatile components of ‘Sachinoka’ strawberry and its somaclonal mutant ‘Mixue’ fruit at four developmental stages. WT represents cultivated strawberry ‘Sachinoka’. *Mut* represents somaclonal mutant ‘Mixue’.

To identify volatile component differences between ‘Sachinoka’ and its somaclonal mutant ‘Mixue’, principal component analysis (PCA) based on the contents of nine categories of volatile components was conducted. The first principal component (PC1) and the second principal component (PC2) accounted for 80.50% and 11.28% of the total variance, respectively (Figure 3). PC1 and PC2 successfully distinguished the contents of volatile components of ‘Sachinoka’ and its somaclonal mutant ‘Mixue’ at four developmental stages. PC1 clearly distinguished the first three stages of fruit development from the red fruit stage. The contents of volatile components of the first three stages of fruit development were close to each other, indicating that these developmental stages had similar volatile components. PC2 successfully distinguished ‘Sachinoka’ and its somaclonal mutant ‘Mixue’ (Figure 3). Benzene and volatile phenols, isoprenoids, acids were the main contributors to the first component, while alcohols, aldehydes and ketones and ethyl esters were the contributors to the second component (Table S7). Significant differences in the contents of volatile components between ‘Sachinoka’ and its somaclonal mutant ‘Mixue’ were found at different fruit development stages, even at the same fruit development stage between ‘Sachinoka’ and its somaclonal mutant ‘Mixue’.

### 2.7. Expression Levels of Anthocyanins-Related Genes

**Figure 4:**
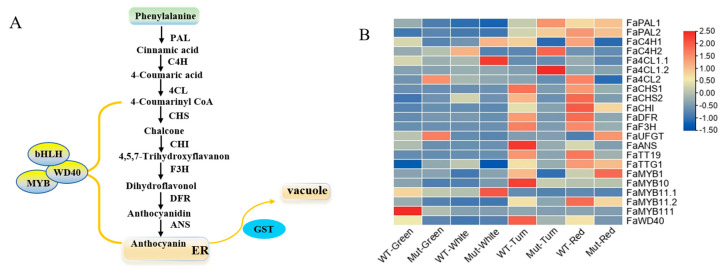
(**A**) Anthocyanin biosynthetic and regulatory pathway. The green box represents the precursor for anthocyanin biosynthesis; the yellow oval represents transcription factors regulating anthocyanins accumulation; the yellow box represents the endoplasmic reticulum and vacuole; the blue oval represents the anthocyanin transport gene. PAL is phenylalanine ammonia-lyase; C4H is cinnamate 4-hydroxylase; 4CL is 4-coumarate-CoA synthase; CHS is chalcone synthase; CHI is chalcone isomerase; F3H is flavanone 3-hydroxylase; DFR is dihydroflavonol-4-reductase; ANS is anthocyanidin synthase; MYB is MYB transcription factor; WD40 is WD-repeat protein; bHLH is basic helix-loop-helix transcription factor. (**B**) Expression levels of anthocyanins-related genes of ‘Sachinoka’ strawberry and its somaclonal mutant ‘Mixue’ fruit at four developmental stages. PAL is phenylalanine ammonia lyase; C4H is cinnamate 4-hydroxylase; 4CL is 4-coumarate-CoA synthase; CHS is chalcone synthase; CHI is chalcone isomerase; F3H is flavanone 3-hydroxylase; DFR is dihydroflavonol-4-reductase; UFGT is UDP glucose flavonoid-3-O-glycosyltranferase; TT19 is transparent testa 19; TTG1 is testa glabra 1; MYB is MYB transcription factor; WD40 is WD-repeat protein; ANS is anthocyanidin synthase.

To investigate the reason for the white flesh phenotype of the somaclonal mutant ‘Mixue’, we detected the expression of 22 anthocyanins-related genes including 15 structural genes and seven regulatory and transport genes at four developmental stages between ‘Sachinoka’ and its somaclonal mutant ‘Mixue’ (Figure 4A). Seven structural genes including *FaPAL1*, *FaCHS1*, *FaCHS2*, *FaCHI*, *FaDFR*, *FaF3H* and *FaANS* had similar expression patterns in ‘Sachinoka’ and its somaclonal mutant ‘Mixue’. The expression levels of these seven structural genes were significantly increased in turning and red fruits in ‘Sachinoka’ and its somaclonal mutant ‘Mixue’, whilst the expression levels of these seven structural genes in turning and red fruits of somaclonal mutant ‘Mixue’ were significantly lower than those in ‘Sachinoka’. In addition, the expression levels of *FaTT19*, *FaMYB10*, *FaMYB11.2* and *FaWD40* in ‘Sachinoka’ at the turning stage were 6.73, 3.02, 2.48, and 2.52 times higher than those in somaclonal mutant ‘Mixue’, respectively (Figure 4B). The expression of *FaMYB1* in red fruit was remarkably increased in the somaclonal mutant ‘Mixue’ compared with ‘Sachinoka’ (Figure 4B). The differential expression of these key factors involved in regulating and transporting anthocyanins between ‘Sachinoka’ and its somaclonal mutant ‘Mixue’ may be the key reason for the white-flesh phenotype in the red fruit stage of the somaclonal mutant ‘Mixue’.

### 2.8. Expression Levels of Genes Involved in Volatile Components Metabolic Pathway

To explore the reason for the differences in volatile components between ‘Sachinoka’ and its somaclonal mutant ‘Mixue’, we detected the correlation between the expression of volatile-related genes and volatile components in the fruit of ‘Sachinoka’ and its somaclonal mutant ‘Mixue’ at different developmental stages.

#### 2.8.1. Fatty Acid Pathway

**Figure 5:**
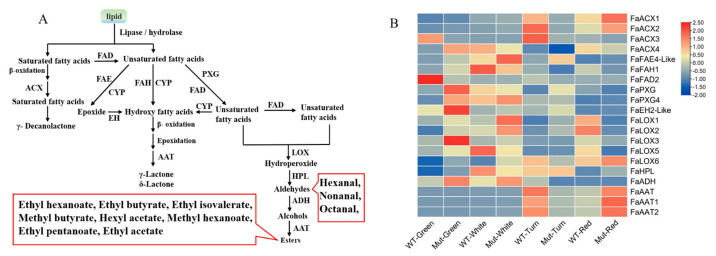
(**A**) Fatty acid pathway. The green box represents the precursor for the fatty acid pathway; the red boxes represent volatile components that play a major role in strawberry aroma (i.e., odor activity values are greater than 1). FAD is fatty acid desaturase; ACX is Acyl-CoA oxidase; FAE is fatty acid elongase; FAH is fatty acid hydroxylase; CYP is cytochrome P450; EH is epoxide hydrolase; PXG is peroxygenase; LOX is lipoxygenase; HPL is hydroperoxide lyase; ADH is alcohol dehydrogenase; AAT is alcohol acyltransferase. (**B**) Expression analysis of biosynthetic genes involved in the fatty acid pathway of ‘Sachinoka’ strawberry and its somaclonal mutant ‘Mixue’ fruit at four developmental stages. FAD is fatty acid desaturase; ACX is Acyl-CoA oxidase; FAE is fatty acid elongase; FAH is fatty acid hydroxylase; EH is epoxide hydrolase; PXG is peroxygenase; LOX is lipoxygenase; HPL is hydroperoxide lyase; ADH is alcohol dehydrogenase; AAT is alcohol acyltransferase.

Esters are the characteristic volatile components in strawberry and are the most abundant volatile components in strawberry [21]. There are 131 different types of esters in strawberry, which account for 25–90% of all strawberry volatile components [19]. Therefore, we analyzed the expression of 20 biosynthetic genes in this pathway between ‘Sachinoka’ and its somaclonal mutant ‘Mixue’ at different developmental stages (Figure 5A).

The biosynthetic genes involved in the fatty acid pathway such as *FaLOX* and *FaADH* had a higher expression in the green and white stages of ‘Sachinoka’ and its somaclonal mutant ‘Mixue’ fruits, which may provide sufficient substrates for the formation of esters during the red fruit stage. Interestingly, a large amount of alcohols accumulated in the green, white and turning fruits (Figure 5B). *FaAAT* directly affects the biosynthesis of esters, especially ethyl hexanoate and ethyl isovalerate, which are characteristic aroma components of strawberry and play important roles in the formation of strawberry fruit aroma [23]. The highest expression levels of *FaAAT1* and *FaAAT2* were found in the turning stage of ‘Sachinoka’ fruit, while the highest expression of *FaAAT1* and *FaAAT2* was found in the red stage of the somaclonal mutant ‘Mixue’ fruit. In addition, we also found the transcript levels of *FaAAT1* and *FaAAT2* in the red stage of the somaclonal mutant ‘Mixue’ fruit were significantly increased compared with ‘Sachinoka’ (Figure 5B), which was consistent with ethyl esters with higher content in the red stage of the somaclonal mutant ‘Mixue’ fruit than in ‘Sachinoka’.

#### 2.8.2. Terpenoid Metabolic Pathway

**Figure 6:**
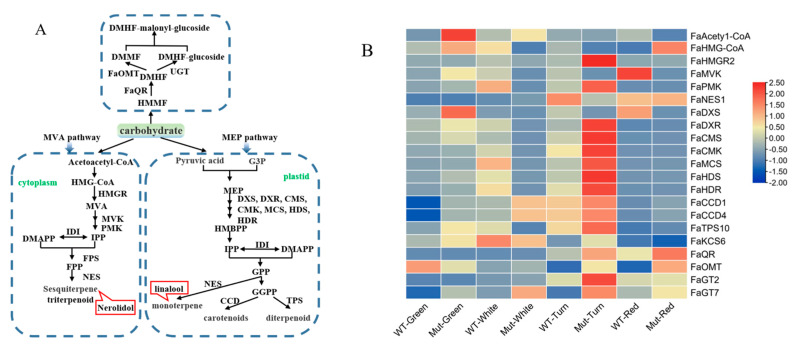
(**A**) Terpenoid metabolic pathway. The green squares represent substrates of the terpenoid metabolic pathway; the red squares represent volatile components that play a major role in strawberry aroma presentation (i.e., OAV values greater than 1). MVA pathway is mevalonate pathway; MEP pathway is 2-C-methyl-D-erythritol 4-phosphate pathway; G3P is glyceraldehyde 3-phosphate; DXS is 1-deoxy-D-xylulose 5-phosphate synthase; DXR is 1-deoxy-D-xylulose 5-phosphate reductoisomerase; FaQR is Fragaria × ananassa quinone oxidoreductase gene; FaOMT is O-methyltransferase gene; UGT is UDP-glucose transporter; MVK is mevalonate kinase; PMK is phosphomevalonate kinase; FPS is farnesyl pyrophosphate synthase; NES is nerolidol synthase; CMS is 4-diphosphocytidyl-2-C-methyl-D-erythritol synthase; CMK is 4-(cytidine 5′-diphospho) -2-C-methyl-D-erythritol kinase; MCS is 2-C-methyl-D-erythritol-2,4-cyclodiphosphate synthase; HDS is 4-hydroxy-3-methylbut-2-en-1-yl diphosphate synthase; HDR is 4-hydroxy-3-methylbut-2-enyl diphosphate reductase; IPP is isopentenyl diphosphate; IDI is Isopentenyl diphosphate isomerase; DMAPP is dimethylallyl diphosphate; FPP is farnesyl diphosphate; GPP is geranyl diphosphate; GGPP is geranylgeranyl diphosphate; HMGR is 3-hydroxy-3-methylglutaryl coenzyme A reductase; CCD is carotenoid cleavage dioxygenase; TPS is terpene synthase. (**B**) Expression analysis of biosynthetic genes involved in terpenoid metableolic pathway of ‘Sachinoka’ strawberry and its somaclonal mutant ‘Mixue’ fruit at four developmental stages: DXS is 1-deoxy- d-xylulose 5-phosphate synthase; DXR is 1-deoxy-D-xylulose 5-phosphate reductoisomerase; FaQR is Fragaria × ananassa quinone oxidoreductase gene; FaOMT is O-methyltransferase gene; UGT is UDP-glucose transporter; MVK is mevalonate kinase; PMK is phosphomevalonate kinase; NES is nerolidol synthase; CMS is 4-diphosphocytidyl-2-C-methyl-D-erythritol synthase; CMK is 4-(cytidine 5′-diphospho)-2-C-methyl-D-erythritol kinase; MCS is 2-C-methyl-D-erythritol-2, 4-cyclodiphosphate synthase; HDS is 4-hydroxy-3-methylbut-2-en-1-yl diphosphate synthase; HDR is 4-hydroxy-3-methylbut-2-enyl diphosphate reductase; IPP is isopentenyl diphosphate; CCD is carotenoid cleavage dioxygenase; TPS is terpene synthase.

Terpenoids are mainly biosynthesized through the mevalonate (MVA) pathway or the 2-C-methyl-D-erythritol 4-phosphate (MEP) pathway. The MVA pathway operates in the cytoplasm and the MEP pathway functions in the plastid. The volatile monoterpenes (C10) and sesquiterpenes (C15) are identified in most fleshy fruits [24]. Therefore, we examined the expression of 21 biosynthetic genes in the terpenoid metabolic pathway between ‘Sachinoka’ and its somaclonal mutant ‘Mixue’ at different developmental stages (Figure 6A).

In the MVA pathway, *FaNES1* is one of the crucial genes involved in sesquiterpenoids biosynthesis [25]. The highest expression of *FaNES1* was found in the turning stage of ‘Sachinoka’ fruit, while the highest expression of *FaNES1* was found in the red stage of the somaclonal mutant ‘Mixue’, which was in accordance with the nerolidol having a 1.69 times higher content in the red fruit stage of the somaclonal mutant ‘Mixue’ than that in ‘Sachinoka’ (Table S3). In addition, *FaNES1* directly regulates the synthesis of the linalool in the MEP pathway [26]. We found that the contents of linalool were almost the same in red fruit stage of ‘Sachinoka’ and the somaclonal mutant ‘Mixue’ (Table S3). The expression of genes related to MEP pathway such as *FaDXR*, *FaCMS*, *FaCMK*, *FaMCS*, *FaHDS* and *FaHDR* reached the highest levels during the turning stage in the somaclonal mutant ‘Mixue’ and the expression levels of these genes in the turning fruit of the somaclonal mutant ‘Mixue’ were significantly higher than those in ‘Sachinoka’. *FaQR*, *FaOMT*, *FaGT2* and *FaGT7* are key genes of the carbohydrate pathway with sugar molecule as the precursors [27]. *FaQR* and *FaOMT* had the highest expression in the red stage of the somaclonal mutant ‘Mixue’ fruit. *FaGT2* and *FaGT7* had the highest expression in the turning stage of the somaclonal mutant ‘Mixue’ fruit. The expression of these genes in the turning and red stages of the somaclonal mutant ‘Mixue’ were significantly increased compared with ‘Sachinoka’ (Figure 6B).

#### 2.8.3. Amino Acid Pathway

**Figure 7:**
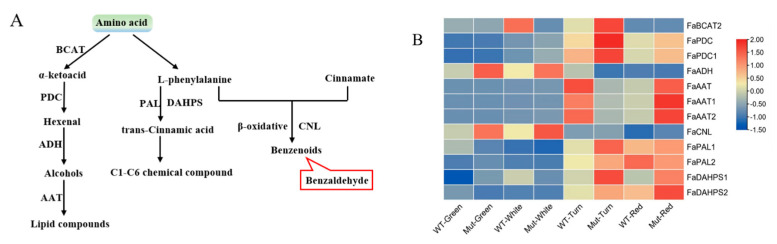
(**A**) Amino acid pathway. The red squares represent volatile components that play a major role in strawberry aroma presentation (i.e., OAV values greater than 1). BCAT is branched-chain aminotransferase; PDC is pyruvate decarboxylase isozyme; ADH is alcohol dehydrogenase; AAT is alcohol acyltransferase; DAHPS is phospho-2-dehydro-3-deoxyheptonate aldolase; PAL is phenylalanine ammonia lyase; CNL is CoA ligase. (**B**) Expression analysis of biosynthetic genes involved in amino acid pathway of ‘Sachinoka’ strawberry and its somaclonal mutant ‘Mixue’ fruit at four developmental stages: BCAT is branched-chain aminotransferase; PDC is pyruvate decarboxylase isozyme; ADH is alcohol dehydrogenase; AAT is alcohol acyltransferase; DAHPS is phospho-2-dehydro-3-deoxyheptonate aldolase; PAL is phenylalanine ammonia lyase; CNL is CoA ligase. ER: endoplasmic reticulum. WT represents cultivated strawberry ‘Sachinoka’. Mut represents somaclonal mutant ‘Mixue’.

In the amino acid pathway, we investigated the expression of 12 genes involved in this pathway between ‘Sachinoka’ and its somaclonal mutant ‘Mixue’ at different developmental stages (Figure 7A). Higher expression levels of *FaPAL1* and *FaDAHPS* were identified in the turning and red stages of ‘Sachinoka’ and its somaclonal mutant ‘Mixue’ than in the green and white stages (Figure 7B), which may lead to a large amount of the trans-cinnamic acid accumulation. In addition, a higher expression of *FaCNL* was found in the green and white fruits of ‘Sachinoka’ and its somaclonal mutant ‘Mixue’, which was consistent with the accumulation of benzenoids components in the green and white fruits of ‘Sachinoka’ and its somaclonal mutant ‘Mixue’ (Figure 7B). In particular, the content of methyl salicylate and phenylethyl alcohol of the somaclonal mutant ‘Mixue’ were 19.19, 9.86 times higher in the green fruit and 2.72, 1.16 times higher in the white fruit compared with ‘Sachinoka’(Table S3). In addition, the transcript level of *FaCNL* was significantly lower in the green and white fruits of ‘Sachinoka’ compared with its somaclonal mutant ‘Mixue’, which may be associated with an increased content of benzaldehyde in the green fruit of somaclonal mutant ‘Mixue’ compared with ‘Sachinoka’ (Figure 2A,B).

## 3. Discussion

Somatic variation is the main source of genetic diversity and plant breeding for asexually propagated plants. Somatic mutation in plants may be genetic [28] or epigenetic [29]. Somatic mutants have nearly identical genetic backgrounds to the parents and are considered as ideal genetic material for studying new traits of mutation [28]. In this study, a somaclonal mutant ‘Mixue’ was identified in the progeny of a micropropagated plant of ‘Sachinoka’. We explored the reasons for the white flesh and stronger aroma of the somaclonal mutant ‘Mixue’ compared with ‘Sachinoka’.

### 3.1. The Reason for White Flesh of Somaclonal Mutant ‘Mixue’

The Somaclonal mutant ‘Mixue’ with white flesh in the red fruit stage was a somaclonal variation of ‘Sachinoka’ strawberry. The white flesh of the red fruit stage of somaclonal mutant ‘Mixue’ resulted from the significantly reduced accumulation of Cy3G and Pg3G in the somaclonal mutant ‘Mixue’ (Figure 1B). The expression of anthocyanin biosynthesis genes *FaPAL*, *FaCHS*, *FaCHI*, *FaDFR*, *FaF3H* and *FaANS* were significantly down-regulated in the turning and red stages of the somaclonal mutant ‘Mixue’ compared with ‘Sachinoka’. Further studies on anthocyanin-related transcription factors and transporter revealed that the expression levels of *FaMYB10*, *FaWD40*, *FaMYB11.2*, and *FaTT19* were significantly reduced in the somaclonal mutant ‘Mixue’ during the turning stage relative to ‘Sachinoka’, while transcript level of FaMYB1 was significantly increased in the red fruit stage of the somaclonal mutant ‘Mixue’ compared with ‘Sachinoka’ (Figure 4B). MYB10 is considered as a critical positive regulator in apple [30], pear [31,32] and strawberry [33,34] for controlling the accumulation of anthocyanins. The lack of anthocyanins in fruit from woodland and cultivated strawberry is mainly derived from the variation in the coding region of *MYB10*, such as a single amino acid change (G35C), 1 or 8 nucleotide insertion, long terminal repeats of transposable element (LTR-TE) insertion [35,36]. Thus, we detected the coding region of *MYB10* between ‘Sachinoka’ and its somaclonal mutant ‘Mixue’. We found the 35th nucleotide in the first exon of the somaclonal mutant ‘Mixue’ was identical to ‘Sachinoka’ and the MYB10 amino acids in the somaclonal mutant ‘Mixue’ did not result in a frameshift and a premature stop codon (Figure S2A). These results indicate that the white flesh of somaclonal mutant ‘Mixue’ is not attributed to the variation in the MYB10 coding region. Some literatures have indicated that deletion or insertion in the strawberry *MYB10* promoter may alter the expression of MYB10, which in turn affects the expression of anthocyanins biosynthetic genes [35,37]. In this study, we also found that the expression of FaMYB10 was significantly down-regulated in the somaclonal mutant ‘Mixue’ (Figure 4B). Moreover, we tested the *FaMYB10* promoter between ‘Sachinoka’ and its somaclonal mutant ‘Mixue’ and found there were no large insertions or deletions in the promoter of ‘Sachinoka’ and the somaclonal mutant ‘Mixue’ and the promoter sequence similarity between ‘Sachinoka’ and its somaclonal mutant ‘Mixue’ was 99.26% (Figure S2B). These results suggest that the white flesh of the somaclonal mutant ‘Mixue’ is associated with a lower expression of *FaMYB10* and the lower expression of *FaMYB10* in the somaclonal mutant ‘Mixue’ was different from the FaMYB10 promoter variations affecting the expression of FaMYB10 [35,36,37]. DNA methylation regulates the expression of *MYB10*, which in turn modulates the biosynthesis of anthocyanins and affects the fruit color in pear [12,31,38]. Further work will be required to better understand whether the different expression patterns of *FaMYB10* in ‘Sachinoka’ and its somaclonal mutant ‘Mixue’ are related to promoter methylation. *MYB1* is an important negative regulator of anthocyanin biosynthesis in strawberry [39]. We found the coding region of *FaMYB1* was identical between ‘Sachinoka’ and its somaclonal mutant ‘Mixue’ (Figure S3A). Gene expression analysis indicated that the expression of *FaMYB1* was markedly increased in the red fruit of the somaclonal mutant ‘Mixue’ compared with ‘Sachinoka’ (Figure 4B). The promoter sequence similarity of *FaMYB1* between ‘Sachinoka’ and its somaclonal mutant ‘Mixue’ was 99.55% (Figure S3B). FaWD40 can form a ternary complex with FaMYB10, FabHLH33 which regulates the expression of downstream anthocyanins structural genes [40]. In this study, we also found that *FaWD40* was significantly down-regulated in the turning and red fruit stages of the somaclonal mutant ‘Mixue’. *FaWD40* may interact with *FaMYB10* and *FabHLH33* to affect the expression of downstream anthocyanins structure genes. The expression patterns of *FaMYB11.2* and *FaTT19* were consistent with *FaMYB10* and *FaWD40*, which had significantly lower expression in the somaclonal mutant ‘Mixue’ fruits during the turning and red stages compared with ‘Sachinoka’ (Figure 5B). *FaMYB9* and *FaMYB11* encode two R2R3-MYB transcription factors which are homologs of Arabidopsis *AtTT2*, which can restore the *tt2-1* mutant phenotype and produce brown seeds [41]. Arabidopsis TT19 is localized on the cytoplasm and vacuole and encodes a glutathione-*S*-transferase (GST). Sun et al. found that *tt19-7* mutants hardly accumulate anthocyanins [42]. Gao et al. knocked out the *RAP* gene (Reduced Anthocyanins in Petioles) in octoploid strawberry using CRISPR/Cas9, resulting in a white-fruited phenotype [43]. Therefore, a lower expression of *FaMYB11.2* and *FaTT19* during the turning and red stages in the somaclonal mutant ‘Mixue’, may lead to a reduction in anthocyanin or proanthocyanidin accumulation.

These results have demonstrated that the differential expression levels of *FaMYB10*, *FaWD40*, *FaMYB11.2*, *FaTT19* and *FaMYB1* in ‘Sachinoka’ and its somaclonal mutant ‘Mixue’ were correlated with the white flesh of the somaclonal mutant ‘Mixue’ fruit. Further study will be needed to determine whether methylation or large transposon, retrotransposon insertion exist in the promoter of these key factors.

### 3.2. The Reasons for Stronger Aroma in Somaclonal Mutant ‘Mixue’ than ‘Sachinoka’

In the last 30 years, the main goal of strawberry breeding has been to produce cultivars with high-yield and large fruit, but the improvement of fruit quality has been neglected [44,45]. During fruit development and ripening, the types and contents of strawberry volatile components are one of the most critical factors to determine the quality of the strawberry [46]. The changes in strawberry fruit quality result from changes in gene expression and enzymatic activity during strawberry fruit development and ripening [47,48]. The content of volatile components in the fruits of ‘Sachinoka’ and its somaclonal mutant ‘Mixue’ gradually increased with the development of fruit. Among them, the contents of acids, acetate, ethyl esters, other esters, benzene and volatile phenols, isoprenoids and furans reached their peak in the red fruit stage (Figure 2D). In addition, ripening fruit of the somaclonal mutant ‘Mixue’ had a stronger aroma than ‘Sachinoka’. The possible reasons are as follows: (1) During the red fruit stage, the content of ethyl hexanoate in the somaclonal mutant ‘Mixue’ was significantly increased than that in ‘Sachinoka’ and ethyl isovalerate had the same trend with ethyl hexanoate (Table S3), which may be related to the significantly increased expression of *FaAAT* in the somaclonal mutant ‘Mixue’ at the red fruit stage compared with ‘Sachinoka’ (Figure 5B). (2) Nerolidol was a unique volatile component in the turning stage of the somaclonal mutant ‘Mixue’ fruit and the content of nerolidol in the somaclonal mutant ‘Mixue’ was significantly higher than that in ‘Sachinoka’ during the red stage (Table S3). Although terpenoids account for less than 10.0% of total strawberry volatile components, the threshold of nerolidol is very low [18], which leads to the high OAVs for nerolidol and has a great contribution to fruit aroma in strawberry. The OAVs in the somaclonal mutant ‘Mixue’ was 1.69 times higher than that in ‘Sachinoka’, which laid the foundation for the stronger aroma in the red fruit stage of the somaclonal mutant ‘Mixue’ than that in ‘Sachinoka’ (Table 1). *FaNES1* is a key gene for the biosynthesis of linalool and nerolidol in strawberry [25]. Increased expression of *FaNES1* in the red fruit stage of the somaclonal mutant ‘Mixue’ may be associated with a higher content of nerolidol compared with ‘Sachinoka’ (Figure 6B). (3) In the amino acid pathway, the content of benzaldehyde in the green fruit increased by 1.40 times in the somaclonal mutant ‘Mixue’ compared with ‘Sachinoka’, which may result from the increased expression of *FaCNL* in the somaclonal mutant ‘Mixue’ than that in ‘Sachinoka’ (Figure 7B). In addition, the content of benzenol in the somaclonal mutant ‘Mixue’ was 1.60 times higher than that in ‘Sachinoka’ during the turning stage (Table S3), which may be related to the significantly increased expression of *FaPAL1* and *FaDAHPS* in the somaclonal mutant ‘Mixue’ than in ‘Sachinoka’ during the turning stage (Figure 7B). (4) Furanones such as HDMF and its derivatives are characteristic volatile components in strawberry. DMHF is the unique and most essential volatile component in strawberry [49]. Although the content of furanones in strawberries are low, it has a very low threshold [15]. Therefore, furanones have a great impact on the aroma of strawberry. *FaQR* is the key gene for the biosynthesis of HDMF. *FaERF9* interacts with *FaMYB98* to form the ERF-MYB complex, which can activate the promoter of *FaQR* and promote HDMF biosynthesis [25]. *FaOMT* encodes the O-methyltransferase which converts HDMF to DMMF [27]. HDMF can also be metabolized to HDMF-glucoside by a UGT and further malonylated into HDMF malonyl-glucoside in strawberry fruit [25]. In this study, the transcript level of *FaQR* during the turning and red stages was significantly increased in the somaclonal mutant ‘Mixue’ than that in ‘Sachinoka’. During the turning stage of ‘Sachinoka’ and its somaclonal mutant ‘Mixue’, the expression levels of *FaQR* was 2.72 times higher relative to ‘Sachinoka’. In the red fruit stage, the expression level of *FaQR* was 2.04 times higher relative to ‘Sachinoka’. Interestingly, *FaOMT* had the same expression pattern with *FaQR* during the turning and red stages in ‘Sachinoka’ and its somaclonal mutant ‘Mixue’ fruits. The expression levels of *FaOMT* in the turning fruit stage was 7.64 times higher relative to ‘Sachinoka’, 15.56 times higher relative to ‘Sachinoka’ fruit during the red stage. In addition, the expression level of *FaGT2* and *FaGT7* in the somaclonal mutant ‘Mixue’ were 2.63 and 8.18 times higher compared with ‘Sachinoka’ during the turning stages, respectively (Figure 6B). However, furanone was not detected in ‘Sachinoka’ and its somaclonal mutant ‘Mixue’ in this study, which may be associated with the very low furanone content in strawberry or inappropriate detection method for furanone. Together, the expression levels of *FaQR*, *FaOMT*, *FaGT2* and *FaGT7* were significantly up-regulated in the somaclonal mutant ‘Mixue’ compared with ‘Sachinoka’ (Figure 7B), which may lead to higher HDMF, DMMF, and DMMF-glucoside contents in the somaclonal mutant ‘Mixue’. Further work will be required to compare the content of furanones in ‘Sachinoka’ and its somaclonal mutant ‘Mixue’.

**Figure 8:**
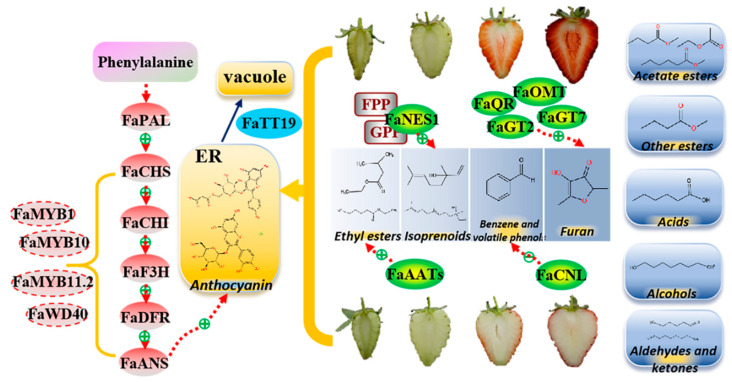
Proposed model for the differences in anthocyanins and volatile components in the fruit of ‘Sachinoka’ strawberry and its somaclonal mutant ‘Mixue’. ER: endoplasmic reticulum. Red ovals are structural genes of the anthocyanin biosynthetic pathway; red dashed ovals are key transcription factors regulating anthocyanins accumulation; blue oval is anthocyanins transport gene; green ovals are key genes in the volatile pathway; gray squares are substrates of the enzymes; blue squares are nine categories of volatile components in strawberry.

In conclusion, the contents of Cy3G and Pg3G were significantly increased in the red fruit stage of ‘Sachinoka’ compared with its somaclonal mutant ‘Mixue’ and the white flesh of the somaclonal mutant ‘Mixue’ may result from the differentially expressed factors involved in anthocyanins accumulation, such as *FaMYB10*, *FaMYB11*.2, *FaWD40*, *FaMYB1*, and *FaTT19*. There were significant increases in the characteristic strawberry volatile components in the red fruit of ‘Mixue’, such as nerolidol, benzaldehyde, ethyl hexanoate, ethyl isovalerate, which led to enhanced volatiles in the somaclonal mutant ‘Mixue’ and might result from the up-regulated expression of *FaNES1*, *FaCNL* and *FaAATs* in ‘Mixue’ compared with ‘Sachinoka’. In addition, the significant up-regulation expression of *FaQR*, *FaOMT*, *FaGT2* and *FaGT7* in the somaclonal mutant ‘Mixue’ during the turning and red fruit stages may result in accumulating more furanones in the somaclonal mutant ‘Mixue’ (Figure 8). These results provide some insights for the somaclonal mutant ‘Mixue’ with white flesh and stronger aroma compared with ‘Sachinoka’.

## 4. Materials and Methods

### 4.1. Plant Material

The materials used in this study were cultivated strawberry (*Fragaria × ananassa* Duch.) cultivars ‘Sachinoka’ and ‘Mixue’. ‘Mixue’ is a somaclonal mutant produced from the tissue culture of apices of runner tips from ‘Sachinoka’. The somaclonal mutant ‘Mixue’ was observed in the field for 3 years and its phenotype was stable. ‘Sachinoka’ and its somaclonal mutant ‘Mixue’ were grown in the greenhouse of Shenyang Agricultural University, China. For different developmental stages of ‘Sachinoka’ and its somaclonal mutant, fruit at 8 days (green stage), 16 days (white stage), 24 days (turning stage) and 32 days (ripening stage) after pollination were collected for HPLC, HS-SPME-GC-MS and RT-qPCR analysis, respectively.

### 4.2. HPLC Analysis

The red stage of ‘Sachinoka’ and its somaclonal mutant ‘Mixue’ fruits were harvested and immediately frozen in liquid nitrogen. Twenty fruits from twenty plants were mixed and ground to fine powder. Some 0.2 g of the powder was added into 2 mL methanol with 1% (v/v) hydrochloric acid and extracted at 4°C for 48 h under dark condition. Centrifugated the extracts at 13,000 rpm for 20 min and collected the supernatant. The residues were re-soaked with 2 mL methanol containing 1% hydrochloric acid and the supernatant collected. Then, hydrochloric acid was added to 5 mL.

The contents of anthocyanins in strawberry fruit were analyzed with the Agilent 1260 Infinity HPLC system equipped with G1314B variable wavelength UV detector (VWD) (Agilent Technologies, Santa Clara, CA, USA). Cyanidin-3-*O*-glucoside (Cy3G) and pelargonidin-3-*O*-glucoside (Pg3G) were purchased from Yuanye Bio-Technology Co., Ltd. (Shanghai, China). These standards were dissolved in methanol to 100 μg/mL. The chromatographic column was Intertsil ODS-35 UM (4.6 mm × 250 mm, 5 µm) and Cy3G and Pg3G were detected at 520 nm. The column temperature was 30°C. The mobile phase A was formic acid dissolved in ultrapure water (1/9, *v*/*v*) and mobile phase B was 10% formic acid dissolved in 90% acetonitrile (*v*/*v*). The injection volume was 10 μL. The flow rate was set at 1 mL/min and the running time was 75 min. Linear gradient elution was used (Table S1). Compositions were distinguished by comparing their retention times with standards under the same condition.

### 4.3. HS-SPME-GC-MS Analysis

The extraction of volatile components from fruit samples was performed following methods previously described un [50]. Weighed 50 g of fruit sample, added 0.5 g of D-gluconolactone and 1 g PVPP (polyvinylpolypyrrolidone), ground into powder. Placed the powder in a 50 mL centrifuge tube, extracted at 4 °C for 4 h, and then centrifuged at 4 °C and 8000 rpm for 10 min to derive strawberry-clarified juice for solid phase microextraction (SPME) and gas chromatography-mass spectrometry (GC-MS) analysis. Extracted the volatile components from strawberry-clarified juice using an SPME fiber coated with 50/30-μm DVB/CAR/PDMS (Superco, Bellefonte, PA, USA) extracts. Divided the aliquot (5 mL) of strawberry clarified juice and 10 μL 4-methyl-2-pentanol (1.002 mg/mL water, internal standard) were mixed in a 15 mL closed vial containing a magnetic stirrer. After 30 min of equilibration at 40 °C, the sample was extracted with SPME fiber (treated at 270 °C for 1 h before use) for 30 min and continuously heated and stirred. The separation and identification of the volatile components were based on Agilent 6890 GC coupled with an Agilent 5975 MS and 60 m × 0.25 mm id HP-INNOWAX capillary column with 0.25 μm film thickness (J&W Scientific, Folsom, CA, USA). The GC-MS in this study was based on methods detailed previously [51]. Under the same chromatographic conditions, the retention index was calculated using C7-C24 n-alkane series (Supelco, Bellefonte, PA, USA). The retention index was based on the reference standard and the mass spectrum matching in the NIST 05 library of standards. When reference standards were not available, the preliminary identification was carried out based on the mass spectrum matching in the NIST 08 library and the identification of retention index was through comparison with the NIST standard reference database (NIST Chemistry WebBook. Available online: https://webbook.nist.gov/chemistry/ (accessed on 14 December 2022).

Take 4-methyl-2-pentanol as the internal standard (add 10 μL of 1.002 mg/mL internal standard aqueous solution to each sample and standard solution) to quantitatively analyze the determined compounds. Two synthetic substrates containing 1% and 11% (v/v) ethanol were prepared in distilled water (having 3.0 g/L malic acid and pH 3.6). Volatile standard solution was dissolved in a 1% and 11% synthetic matrix, and the concentration was usually the concentration of strawberry-clarified juice, respectively. Then the volatile standards were extracted and analyzed under the same conditions as strawberry-clarified juice samples. The quantitative data of volatile components were calculated by the following formula:

The concentration of analyte = (the area of analyte/the area of 4-methyl-2-pentanol) × concentration to 4-methyl-2-pentanol

### 4.4. Quantitative Real-Time PCR

The four developmental stage fruits (more detailed descriptions were shown in Plant material) of ‘Sachinoka’ and its somaclonal mutant ‘Mixue’ fruits were harvested and immediately frozen in liquid nitrogen. At each stage of fruit development, twenty fruits from twenty plants were mixed and ground to fine powder, respectively. Total RNA from four developmental stage fruits of ‘Sachinoka’ and its somaclonal mutant ‘Mixue’ was extracted using the CTAB method. Then, cDNA was synthesized based on PrimeScript TM RT reagent Kit (TaKaRa, Dalian, China). Quantitative real-time RT-PCR (RT-qPCR) was conducted using UltraSYBR Mixture (CWBio, Beijing, China) with quantitative primers (Table S2). RT-qPCR was performed with the QuantStudio TM 6 Flex Real-Time PCR System including 5 μL UltraSYBR Mixture, 0.2 μM quantitative primers, 0.5 μL diluted cDNA and 3.5 μL ddH_2_O. The first step of thermal cycling denaturation was 95 °C for 10 min, followed by 40 cycles for denaturation at 95 °C for 15 s and annealing/extension at 60 °C for 1 min. The relative mRNA levels were calculated using the 2^−ΔΔCt^ method. All data were normalized with the expression level of the 26S and then normalized with the expression of ‘Sachinoka’. Each sample was analyzed in triplicate with three biological replicates.

### 4.5. Statistical Analysis

SPSS 18.0 was applied for statistical analysis (SPSS Inc., USA). An independent *t*-test was used to evaluate the significant difference between the two treatments in the nine categories of volatile components (* *p* < 0.05; ** *p* < 0.01). A one-way analysis of variance (ANOVA) was used to analyze the content of volatile substances at different developmental stages employing Duncan’s multiple range tests at a level of *p* < 0.05. The volatile components and OAVs were presented as the mean ± SD of triplicate measurements. Origin 2021 (OriginLab Inc., Northampton, MA, USA) was used to visualize the data.

## 5. Conclusions

The white flesh in the ripening fruit of ‘Mixue’ was due to the lower expression of *FaMYB10*, *FaMYB11.2*, *FaWD40* and *FaTT19* and higher expression of *FaMYB1* genes. Fifteen volatile components in the ripening fruit of ‘Mixue’ were significantly increased compared with ‘Sachinoka’, such as nerolidol, benzaldehyde, ethyl hexanoate, ethyl isovalerate, which led to an enhanced aroma in ‘Mixue’. The enhanced aroma in ‘Mixue’ was related to the up-regulated expression of *FaNES1*, *FaCNL* and *FaAATs*. These results provide useful information on the effect of somaclonal variation on metabolic components of strawberry fruit and lay the foundation for the improvement in quality of strawberry.

## Figures and Tables

**Table 1:** The odor activity values (OAVs) of partial volatile components in ‘Sachinoka’ strawberry and its somaclonal mutant ‘Mixue’.

Volatile Components	Classification	Olfactory Threshold (mg × kg^−1^)	WT Green	WT White	WT Turn	WT Red	Mut Green	Mut White	Mut Turn	Mut Red
Ethyl hexanoate	Ethyl esters	0.0003 ^a,d^	0.00	0.00	0.00	6391.77	0.00	0.00	5.53	10,844.39
Ethyl butyrate	Ethyl esters	0.001 ^b,d,e^	0.00	0.00	1.29	3105.80	3.76	1.07	6.43	3142.38
Linalool	Isoprenoids	0.001 ^b,c,d^	2.08	4.95	11.68	939.29	8.20	6.99	84.86	927.83
Ethyl isovalerate	Ethyl esters	0.002 ^e^	0.00	0.00	0.00	57.98	0.00	0.00	0.00	128.67
Methyl butyrate	Other esters	0.01 ^b,c^	0.43	0.47	0.93	139.70	0.00	0.56	6.34	120.41
Hexyl acetate	Acetate esters	0.002 ^a,d,e^	0.20	10.70	2.06	168.27	1.12	2.07	15.46	49.47
Methyl hexanoate	Other esters	0.087 ^a,d^	0.00	0.00	0.03	42.18	0.00	0.00	0.68	25.92
Ethyl pentanoate	Ethyl esters	0.0015 ^c^	0.27	0.24	0.24	9.76	0.83	0.42	0.36	10.94
Hexanal	Aldehydes and ketone	0.1 ^b^	2.32	3.34	2.94	6.56	9.20	2.99	3.97	6.50
Octanal	Aldehydes and ketone	0.001 ^e^	1.13	0.80	0.72	10.57	1.34	0.93	1.21	7.26
Nonanal	Aldehydes and ketone	0.001 ^e^	0.37	0.42	0.37	1.59	1.69	0.48	0.75	5.99
Nerolidol	Isoprenoids	0.1 ^b^	0.00	0.00	0.00	1.65	0.00	0.00	0.01	2.80
Ethyl Acetate	Acetate esters	1 ^bc^	0.01	0.01	0.01	2.11	0.02	0.01	0.02	1.70
Benzaldehyde	Benzene and volatile phenols	0.35 ^e^	0.01	0.01	0.01	0.84	0.01	0.01	0.01	1.10
Hexanol	Alcohols	0.1 ^b^	0.06	1.61	0.37	0.40	0.36	0.27	0.66	0.22
cis-3-Hexenol	Alcohols	0.03^c^	0.05	0.29	0.11	0.04	0.70	0.09	0.22	0.03
trans-2-Hexenol	Alcohols	1 ^b^	0.00	0.00	0.00	0.01	0.00	0.00	0.00	0.01
1-Octanol	Alcohols	0.11 ^e^	0.01	0.01	0.02	0.04	0.02	0.01	0.02	0.03
Acetic acid	Acid	100 ^b^	0.00	0.00	0.00	0.00	0.00	0.00	0.00	0.00
Butanoic acid	Acid	1 ^b^	0.00	0.00	0.00	0.21	0.00	0.00	0.00	0.18
Hexanoic acid	Acid	10 ^b^	0.00	0.00	0.00	0.81	0.00	0.00	0.01	0.57
Octanoic acid	Acid	0.91 ^c^	0.00	0.00	0.00	0.95	0.00	0.00	0.00	0.76
trans-3-Hexenyl acetate	Acetate esters	0.016 ^c^	0.02	0.19	0.10	0.84	0.31	0.11	0.62	0.74
trans-2-Hexenyl acetate	Acetate esters	0.21 ^c^	0.00	0.20	0.05	0.47	0.02	0.05	0.43	0.30
Butyl butylate	Other esters	0.11 ^c^	0.00	0.00	0.00	0.30	0.00	0.00	0.00	0.34
Hexyl butyrate	Other esters	0.25 ^a,e^	0.00	0.00	0.00	0.01	0.00	0.00	0.00	0.01
Benzyl acetate	Benzene and volatile phenols	0.75 ^e^	0.00	0.00	0.00	0.10	0.00	0.00	0.00	0.02
Methyl salicylate	Benzene and volatile phenols	0.004 ^c^	0.00	0.01	0.01	0.49	0.07	0.02	0.08	0.52
Benzyl alcohol	Benzene and volatile phenols	0.62 ^c^	0.00	0.00	0.00	0.01	0.01	0.00	0.01	0.01

The letters (a–e) in this table indicated published literatures to obtain the threshold values of aroma substances in strawberry. ^a^ [21]; ^b^ [17]; ^c^ [20]; ^d^ [18]; ^e^ [19]; WT represents cultivated strawberry ‘Sachinoka’. Mut represents somaclonal mutant ‘Mixue’.

## Data Availability

The data presented in this study are available on request from the corresponding author.

## Supplementary Materials

The following supporting information can be downloaded at: https://www.mdpi.com/article/10.3390/plants12010082/s1, Figure S1: Phenotype of ‘Sachinoka’ (WT) and its somaclonal mutant ‘Mixue’(*Mut*) at different developmental stages; Figure S2: The alignment of coding region of Fa*MYB10* and promoter sequence alignment of *FaMYB10* of ‘Sachinoka’ (WT) and its somaclonal mutant ‘Mixue’(*Mut*); Figure S3: The alignment of coding region of *FaMYB1* and promoter sequence alignment of *FaMYB1* of ‘Sachinoka’ (WT) and its somaclonal mutant ‘Mixue’(*Mut*); Table S1: Linear gradient elution of anthocyanins; Table S2: Primers used for gene expression analysis; Table S3: Significant differences in volatile components contents between ‘Sachinoka’ strawberry and somaclonal mutant ‘Mixue’; Table S4: The proportion of each major component in the four periods in ‘Sachinoka’ strawberry (WT) and its somaclonal mutant ‘Mixue’ (*Mut*); Table S5: The unique aroma substances in the ‘Sachinoka’ strawberry; Table S6: The unique aroma substances in somaclonal mutant ‘Mixue’; Table S7: Principal component load matrix of volatile components.
