# Supplementary material for: An Integrated Metabolomic and Gene Expression Analysis of ‘Sachinoka’ Strawberry and Its Somaclonal Mutant Reveals Fruit Color and Volatiles Differences
Source: Plants (Basel). 2022 Dec 23;12(1):82. doi: 10.3390/plants12010082 (PMC9824559; doi:10.3390/plants12010082)
Supplement: Supplementary file 1 [file plants-12-00082-s001.zip › plants-2011282-supplementary.pdf]

Figure S1

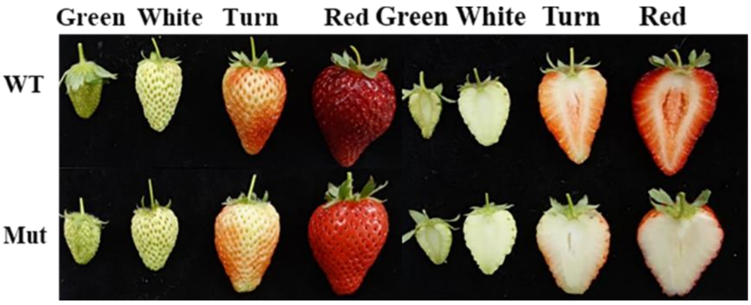

Figure S1. Phenotype of ‘Sachinoka’ (WT) and its somaclonal mutant ‘Mixue’(*Mut*) at different developmental stages.

Figure S2

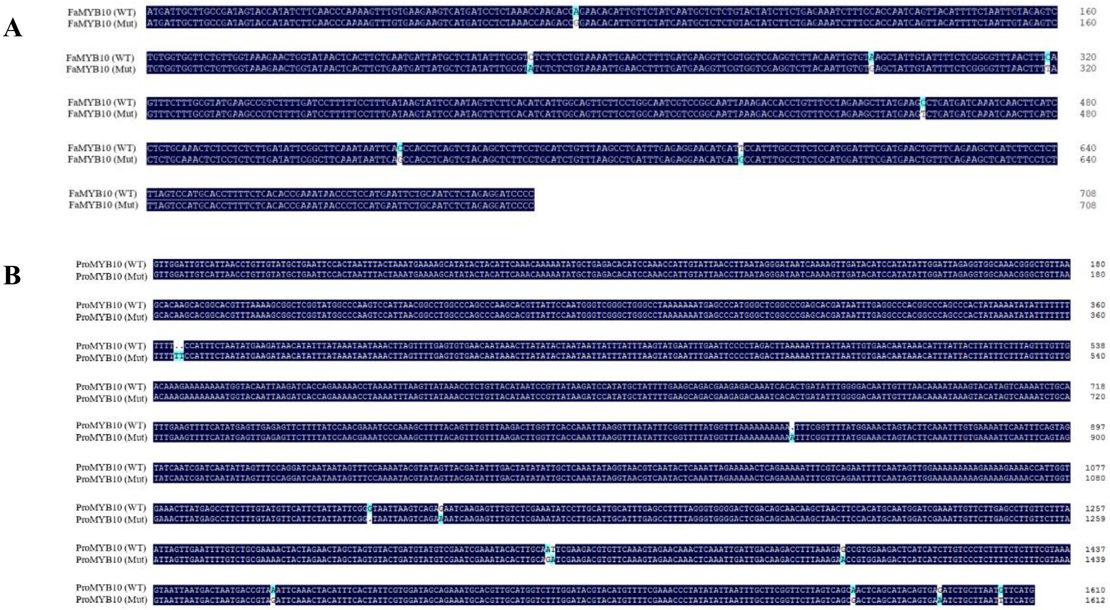

Figure S2. The alignment of coding region of *FaMYB10* (A) and promoter sequence alignment of *FaMYB10* (B) of ‘Sachinoka’ (WT) and its somaclonal mutant ‘Mixue’(*Mut*).

Figure S3

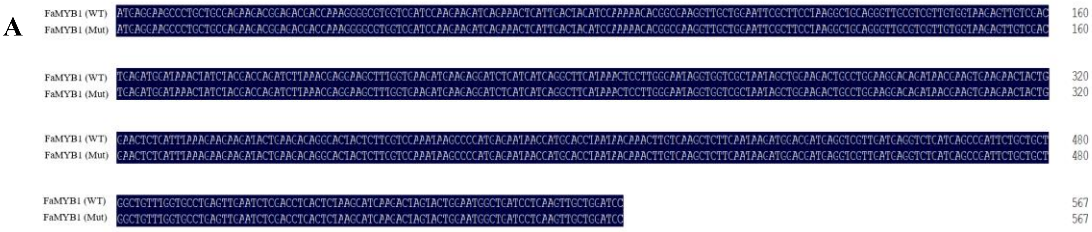

B

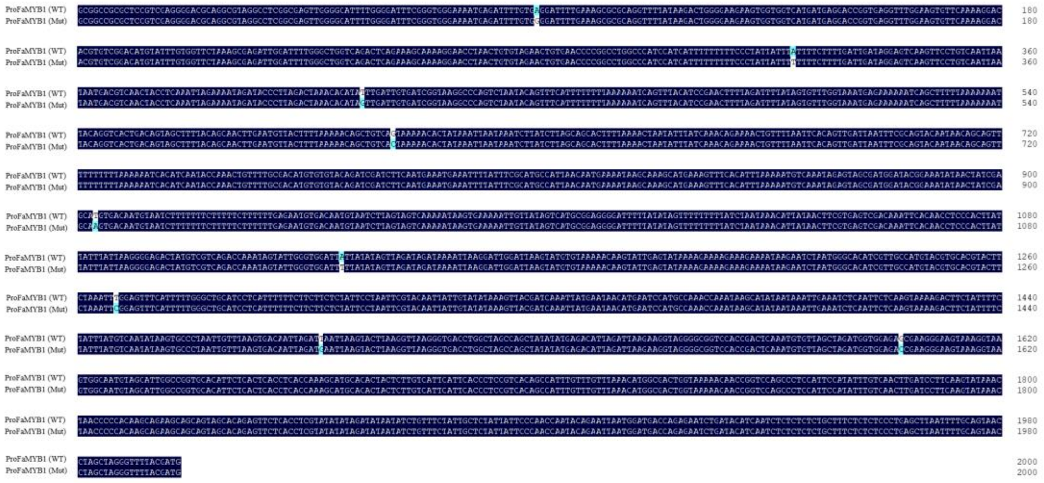

Figure S3. The alignment of coding region of *FaMYB1* (A) and promoter sequence alignment of *FaMYB1* (B) of 'Sachinoka' (WT) and its somaclonal mutant 'Mixue'(*Mut*).

SUPPLEMENTAL TABLES

Table S1. Linear gradient elution of anthocyanins

Time (min)	A Value	B Value
0	95%	5%
25	85%	15%
42	78%	22%
60	64%	36%
65	95%	5%
75	95%	5%

Table S2. Primers used for gene expression analysis

Primer	Sequence (5' to 3')
FaMYB10-F	ATGGAGGGTTATTTCGGTGTG
FaMYB10-R	TCATACGTAGGAGATGTTGAC
FaMYB10-Pro-F	CACTGTTGATACATATGCGTTGGATTGTCATTAACCTGTTGT
FaMYB10-Pro-R	TTCAGAATTCGGATCCTCGATGAACTGTTTCAGAAGTTCAT
FaMYB1-F	GCACATATGATGAGGAAGCCCTGCTGCGA
FaMYB1-R	GCAGGATCCAGCAACTTGAGGATCAGCCATT
FaMYB1-Pro-F	CACGGGGGACTCTAGGCGGCCGCGCTCCGTCGAGGGGA
FaMYB1-Pro-R	TTCAGAATTCGGATCCATCGTAAAACCCTAGCTAGGT
FaPAL1-qPCR-F	GCTGAGCAACACAACCAAGA

FaPAL1-qPCR-R	GTCCTCCTGGCCACTTTACT
FaPAL2-qPCR-F	AGTCTCACACTCTGCCTCAC
FaPAL2-qPCR-R	AGGCAAGCAGGGAGTAATGT
FaPAL1.2-qPCR-F	TGGACTACGGCTTCAAAGGT
FaPAL1.2-qPCR-R	TCTTGGTTGTGTTGCTCAGC
FaC4H1-qPCR-F	AGGCCATGGAGTTCAAGTCA
FaC4H1-qPCR-R	GTTTCTTCCAGTGTGCAGGG
FaC4H2-qPCR-F	CTGTCCAATGCAAGATCCGG
FaC4H2-qPCR-R	ACCATGCATTGACAACCACC
Fa4CL1.1-qPCR-F	CATAGGGTTAGTGTTGCGCC
Fa4CL1.1-qPCR-R	CTGCCTCCGTCATCCCATAT
Fa4CL1.2-qPCR-F	GCGAAAACCCGAACCTCTAC
Fa4CL1.2-qPCR-R	CAAGTCGTACTGGTGGAGGT
Fa4CL2-qPCR-F	CTCCACACCTACTGCTTCGA
Fa4CL2-qPCR-R	CTTGAAGATTTCGGAGGCGG
FaCHS1-qPCR-F	GCTTTTGGAATGGCATGAAT
FaCHS1-qPCR-R	ACCCAAGTGTGGGGTCATAA
FaCHS2-qPCR-F	TTCCAAGCTAACGGCTCTGT
FaCHS2-qPCR-R	CTGGAGGACAGAATGCCAAT
FaCHI-qPCR-F	AGGAGGAAGAAGCCTTGGAG
FaCHI-qPCR-R	GAGACACCCCTCTTGTTCCA
FaDFR-qPCR-F	CTGGAGCGATGTCGAATTTT
FaDFR-qPCR-R	AGAGGCGAAAGTCCGGTAAT
FaF3H-qPCR-F	AGGGTGGCTTCATCGTTTC
FaF3H-qPCR-R	GGCTGGGGGCATTTCGGGT
FaUFGT-qPCR-F	GCCTGGATCCGTTGTGTACT
FaUFGT-qPCR-R	CAAGATCAATGGCTGCTGAA
FaANS-qPCR-F	CCTCAAACACCTTCCGACT
FaANS-qPCR-R	CCTCCCTTCTTCTAATCCC
FaTT19-qPCR-F	TCACATTCTTGCTCGACAG
FaTT19-qPCR-R	ATTGATCCACCAGTGCCTTC
FaTTG1-qPCR-F	CTACCCTCTCTACGCCATGG
FaTTG1-qPCR-R	AAGGAGAGAATGTCGACCCG
FaMYB1-qPCR-F	TTGCGTCGTTGTGGTAAGAG
FaMYB1-qPCR-R	TCTGTCCTTCCAGGCAGTCT
FaMYB10-qPCR-F	GAGAAGGCAAATGGCATCAT
FaMYB10-qPCR-R	GCAATCTTCCGGCAATTAAA
FaMYB11.1-qPCR-F	AGGGATTATGCCATCGTCGT
FaMYB11.1-qPCR-R	ACCCGAGTTTCCAGTCTTGT
FaMYB11.2-qPCR-F	CTGAGGCCTATGTACCAGCA
FaMYB11.2-qPCR-R	GCGGCAGAGTAGTTGACATG
FaMYB111-qPCR-F	AACGCAGGTCAAGTGGAAAC
FaMYB111-qPCR-R	GCACACCACCCAACATTCTT
FaWD40-qPCR-F	GCCGTGACAGATTTGCCATA

FaWD40-qPCR-R	TTTGAGGTGCCATCATCCAG
FaAcety1-CoA-qPCR-F	TTGTTGGTTCTGGATGCAGC
FaAcety1-CoA-qPCR-R	GCCATGTCCAGCACCATTAG
FaHMG-CoA-qPCR-F	TGGTTCAACTGCAACCATGT
FaHMG-CoA-qPCR-R	CCTCCATACCTGTGCTCCAT
FaHMGR2-qPCR-F	GAGACGAAGCTGGGAGATTG
FaHMGR2-qPCR-R	ATAGCCCACTGGCATTTCAC
FaMVK-qPCR-F	GGAAGTCAAAGCCAGAGCAC
FaMVK-qPCR-R	GTTGATGGGTTGGGAACATC
FaPMK-qPCR-F	CCGAGGAATCAAATGGAGAA
FaPMK-qPCR-R	CAAAACCACTGCCCACTTTT
FaNES1-qPCR-F	AGTATTGTGTCTGCCCGTGA
FaNES1-qPCR-R	CCTTCTGTACCTAGCTGCGA
FaDXS-qPCR-F	CACAAAGACCACCCACTCCT
FaDXS-qPCR-R	ACAACACCAAGGCTTGATCC
FaDXR-qPCR-F	AGCAAATGAAAAGGCTGTGG
FaDXR-qPCR-R	CGTGCCCACAAATCATAGTG
FaCMS-qPCR-F	AAGTGCCTACAGGAGCAGGA
FaCMS-qPCR-R	ACAGGATGTGGAAGGTGCTC
FaCMK-qPCR-F	AAGTGCCTACAGGAGCAGGA
FaCMK-qPCR-R	CAAAGGGTACAGGTGGAGGA
FaMCS-qPCR-F	CCCAGAACCAAACCCCTACT
FaMCS-qPCR-R	CCACCACACAATGAAGCAAC
FaHDS-qPCR-F	GATAGACGCGCACAGTTTGA
FaHDS-qPCR-R	CTCGCAAATTCAAATGCAGA
FaHDR-qPCR-F	GGTGTTCCGGAAGAATCTCA
FaHDR-qPCR-R	TCTGGCCTCGTAAGCAATCT
FaCCD1-qPCR-F	ATGCTTGGGAGGAAGAGGAC
FaCCD1-qPCR-R	TCTGTGTTGCTAGACCGGTT
FaCCD4-qPCR-F	GCAGGATGTACGGTGAGGAT
FaCCD4-qPCR-R	GATGTCCAGCTCAGACGACA
FaTPS10-qPCR-F	AAGCAAAGGATTGGTTGGTG
FaTPS10-qPCR-R	GTGAAGGCAATGCAGCACTA
FaKCS6-qPCR-F	TACAAGCCCCCTGTTACCTG
FaKCS6-qPCR-R	GGAGTCCATTGCGGAGAATA
FaQR-qPCR-F	AGCTGGTGTGGTGGTAAAGG
FaQR-qPCR-R	CCTTCATGGGCAGTTTCAAT
FaOMT-qPCR-F	GCTACCCGAGAGAACACGAG
FaOMT-qPCR-R	TGCTGTGATTTTGCCATCAT
FaGT2-qPCR-F	TTTTTGGAGAAGGCAGGAGA
FaGT2-qPCR-R	AAATACTTGGCGTCGGTCAC
FaGT7-qPCR-F	GCACCACTCTTCTCCAAAGC
FaGT7-qPCR-R	AGGCAATGAGGTCGATGTTC
FaACX1-qPCR-F	CAAACAAGGTTCACTCGCCA

FaACX1-qPCR-R	CGCCTCCTCGTAAAGTTTCG
FaACX2-qPCR-F	ACTGCTCGATGGGAAAGTGA
FaACX2-qPCR-R	CAGCTCGAGAACTTGCATCC
FaACX3-qPCR-F	TCCATGTTGTTTCAAGCGC
FaACX3-qPCR-R	AGGTGAGGTGGAATGACAGG
FaACX4-qPCR-F	GCAACAAAGGTGGAAGGAGG
FaACX4-qPCR-R	TCCATTTTGAACCATGCGCA
FaFAE4-Like-qPCR-F	AATGGGGTGTAGTGCTGGAG
FaFAE4-Like-qPCR-R	CGACGCCTATCCAATGACTT
FaFAH1-qPCR-F	GCCCCACTTTTATTGCCACA
FaFAH1-qPCR-R	CGACTCACCCTCACCTCTTT
FaFAD2-qPCR-F	GTCCCTTGTTGGTGGTGAAC
FaFAD2-qPCR-R	AAGGCTCCTCTCAACCAGTC
FaPXG-qPCR-F	CTTTGGCAACATCAGCAAAA
FaPXG-qPCR-R	CAAGAGCTCGACATCCAACA
FaPXG4-qPCR-F	TTTCATCAAGCATGCACACA
FaPXG4-qPCR-R	GCTCCCATCGTAGACAGCTC
FaEH2-Like-qPCR-F	GTGCTTTTCCTGCATGGATT
FaEH2-Like-qPCR-R	CCAAAAGGCCAACAACATCT
FaLOX1-qPCR-F	GGGAGTCCAGACAAAGGTGA
FaLOX1-qPCR-R	TTCAAGTTTGCGGCTCTTCC
FaLOX2-qPCR-F	AGAGTGTGCTGACTATCCCG
FaLOX2-qPCR-R	GGAATCCGGTGAGCTCGATA
FaLOX3-qPCR-F	TTGGGAGGCTAAAGGGAGTG
FaLOX3-qPCR-R	AAATTCGTCATCTCGCAGCC
FaLOX5-qPCR-F	GAAGAGCTCCGCAATGAAGG
FaLOX5-qPCR-R	GGCCACATCAGGGTTAGTCT
FaLOX6-qPCR-F	GAACTAGCTAGGCCGGTCAT
FaLOX6-qPCR-R	GCAAGGCTTTCAACTTCCCA
FaHPL-qPCR-F	AACGCATTTGGTGGTTTCTC
FaHPL-qPCR-R	GTTGAGCCGGAGTGTTTCAT
FaADH-qPCR-F	GAACCCCAAAGACCACAAGA
FaADH-qPCR-R	AAGAACAGCAACACCCCAAC
FaBCAT2-qPCR-F	CAACGGCCGGGACTATACTA
FaBCAT2-qPCR-R	AACAGTTTGGGCACCTGTTC
FaPDC-qPCR-F	CACCTCCACCGTTCAAAACT
FaPDC-qPCR-R	GAGGTGGTCAAGAAGGGTCA
FaPDC1-qPCR-F	TGCCTTTTGTGCTGAGATTG
FaPDC1-qPCR-R	TTGCTAGGCCTACGAGGAAA
FaAAT-qPCR-F	TGCTTTTCCCACCGAGAGAT
FaAAT-qPCR-R	AAACCAGTGACGGCATGAAC
FaAAT1-qPCR-F	GCAATCGGTGTCTTCGTCTC
FaAAT1-qPCR-R	ATCTCTCGGTGGGAAAAGCA
FaAAT2-qPCR-F	GGCTCTGATGGCATTCTTGG

FaAAT2-qPCR-R	GGACCGTTGCACCATAGTTC
FaCNL-qPCR-F	CAATTACGCAGCTCTCACCC
FaCNL-qPCR-R	GATCATGAGTTTGGCGCCAT
FaDAHPS1-qPCR-F	GACCCGTTTGAGGAGAAGGA
FaDAHPS1-qPCR-R	CGCTCTGCTCTGTGAAATCC
FaDAHPS2-qPCR-F	CATGAGAGTCAAGCTTCCGC
FaDAHPS2-qPCR-R	CGTACCTCAGCCAGAATTGC

Table S3. Significant differences in volatile components contents between ‘Sachinoka’ strawberry and somaclonal mutant ‘Mixue’

		Relative contents							
	RI	WT-Green	WT-White	WT-Turn	WT-Red	Mut-Green	Mut-White	Mut-Turn	Mut-Red
Alcohols									
2-Hexanol	1222	1.0000±0.0000a	1.0000±0.0000a	0.9999±0.0000a	0.9982±0.0020b	1.0000±0.0000a	0.9992±0.0012ab	1.0000±0.0000a	1.0000±0.0009a
Pentyl alcohol	1256	0.0029±0.0003e	0.0056±0.0002bc	0.006.±0.0004b	0.0004±0.0004d	0.0006±0.0007b	0.0004±0.0004d	0.0110±0.0009a	0.0051±0.0004cd
2-Heptanol	1327	0.0034±0.0001d	0.0038±0.0011d	0.0037±0.0005d	0.0861±0.0009a	0.0097±0.0014c	0.0005±.0008d	0.0006±0.0016d	0.0514±0.0033b
cis-2-Pentenol	1309	0.0013±0.0000f	0.0036±0.0002d	0.0033±0.0000d	0.0032±0.0001d	0.0079±0.0005a	0.0024±0.0002e	0.0046±0.0002b	0.0040±0.0004c
Hexanol	1356	0.0060±0.0001e	0.1615±0.0066a	0.0374±0.0014c	0.0401±0.0010c	0.0362±0.0006c	0.0271±0.0003d	0.0656±0.0075b	0.0495±0.0463d
trans-3-Hexenol	1385	0.0000±0.0000e	0.0011±0.0001a	0.0003±0.0001d	0.0006±0.0000c	0.0006±0.0001c	0.0003±0.0000d	0.0007±0.0007c	0.0009±0.0001b
cis-3-Hexenol	1371	0.0015±0.0001f	0.0086±0.0001b	0.0034±0.0000d	0.0011±0.0001f	0.0211±0.0006a	0.0027±0.0001c	0.0065±0.0007d	0.0010±0.0001f
trans-2-Hexenol	1324	0.0025±0.0007cd	0.0014±0.0003d	0.0013±0.0001d	0.0072±0.0021b	0.0042±0.0010c	0.0020±0.0009d	0.0031±0.0011cd	0.0093±0.0004a
cis-2-Hexenol	1398	0.0085±0.0001e	0.1842±0.0048a	0.0476±0.0016c	0.0046±0.0002e	0.0336±0.0004d	0.0303±0.0003d	0.1061±0.0115b	0.0084±0.0003e
1-Heptanol	1460	0.0006±0.0002ef	0.0008±0.0000de	0.0008±0.0001d	0.0024±0.0001a	0.0013±0.0001c	0.0005±0.0000f	0.0009±0.0002d	0.0021±0.0001b
2-Ethyl-1-hexanol	1489	0.0087±0.0001bc	0.0106±0.0006a	0.0107±0.0007a	0.0097±0.0020abc	0.0087±0.0003bc	0.0081±0.0003c	0.0099±0.0010ab	0.0087±0.0005bc
3-Ethyl-4-methylpentanol	1500	0.0001±0.0000e	0.0002±0.0000de	0.0002±0.0000de	0.0005±0.0001c	0.0007±0.0000b	0.0002±0.0000d	0.0005±0.0001c	0.0012±0.0001a
1-Octanol	1557	0.0011±0.0001f	0.0016±0.0001e	0.0019±0.0001d	0.0048±0.0001a	0.0020±0.0001d	0.0014±0.0000ef	0.0023±0.0004c	0.0033±0.0002b
trans-2-Octenol	1616	0.0008±0.0001d	0.0017±0.0001a	0.0014±0.0001b	0.0012±0.0001c	0.0014±0.0001b	0.0008±0.0001d	0.0019±0.0002a	0.0014±0.0000bc
Nonanol	1654	0.0006±0.0001de	0.0006±0.0001de	0.0005±0.0000e	0.0017±0.0001a	0.0010±0.0002c	0.0007±0.0001de	0.0008±0.0002d	0.0013±0.0001b
1-Pentene-3-ol	1162	0.0016±0.	0.0055±0.	0.0059±0.	0.0128±0.	0.0122±0.0	0.0041±0.0	0.0077±0.	0.0069±0.

		0001d	0006bc	0002bc	0034a	004a	007cd	0015b	0029bc
2-methyl-5-Hepten-1-ol	1730	0.0001±0.0000d	0.0001±0.0000d	0.0001±0.0000d	0.0052±0.0001b	0.0005±0.0001c	0.0001±0.0000d	0.0002±0.0000d	0.0055±0.0003a
Acid									
Acetic acid	1449	0.0023±0.0004b	0.0010±0.0002b	0.0014±0.0002b	0.1500±0.0338a	0.0017±0.0006b	0.0008±0.0002b	0.0008±0.0002b	0.1285±0.0131a
Isobutyric acid	1557	0.0002±0.0000c	0.0002±0.0000c	0.0003±0.0001c	0.1490±0.0142a	0.0000±0.0000c	0.0003±0.0000c	0.0008±0.0002c	0.0995±0.0080b
Butanoic acid	1574	0.0001±0.0001c	0.0001±0.0000c	0.0002±0.0001c	0.2138±0.0339a	0.0000±0.0000c	0.0000±0.0000c	0.0006±0.0001c	0.1807±0.0162b
Methylethylacetic acid	1658	0.0001±0.0001c	0.0005±0.0001c	0.0026±0.0011c	1.4204±0.1210a	0.0000±0.0000c	0.0010±0.0002c	0.0159±0.0803c	1.0541±0.0803b
Hexanoic acid	1762	0.0039±0.0028c	0.0054±0.0011c	0.0138±0.0065c	8.1464±1.2719a	0.0027±0.0002c	0.0029±0.0004c	0.0546±0.0112c	5.4748±0.6808b
trans-3-Hexenoic Acid	1909	0.0000±0.0000c	0.0000±0.0000c	0.0000±0.0000c	0.0109±0.0032a	0.0000±0.0000c	0.0000±0.0000c	0.0000±0.0000c	0.0020±0.0040b
trans-2-Hexenoic acid	1933	0.0046±0.0000c	0.0017±0.0001c	0.0012±0.0004c	0.0136±0.0021a	0.0020±0.0001c	0.0008±0.0001c	0.0016±0.0002c	0.0078±0.0011b
Octanoic acid	2052	0.0007±0.0004c	0.0007±0.0002c	0.0007±0.0003c	0.8607±0.1420a	0.0002±0.0000c	0.0003±0.0001c	0.0009±0.0002c	0.6892±0.0928b
Nonanoic acid	2155	0.0006±0.0002c	0.0007±0.0003c	0.0004±0.0002c	0.0029±0.0005a	0.0002±0.0001c	0.0004±0.0001c	0.0003±0.0001c	0.0017±0.0002b
Ethyl esters									
Ethyl propanoate	946	0.0000±0.0000c	0.0000±0.0000c	0.0000±0.0000c	0.1027±0.0006a	0.0000±0.0000c	0.0000±0.0000c	0.0000±0.0000c	0.0603±0.0060b
Ethyl butyrate	1039	0.0000±0.0000b	0.0000±0.0000b	0.0013±0.0001b	3.1058±0.0555a	0.0038±0.0003b	0.0011±0.0058b	0.0064±0.0043b	3.1423±0.1931a
Ethyl 2-methylbutyrate	1062	0.0000±0.0000c	0.0000±0.0000c	0.0000±0.0000c	0.0406±0.0018b	0.0000±0.0000c	0.0000±0.0000c	0.0000±0.0000c	0.0494±0.0032a
Ethyl isovalerate	1093	0.0000±0.0000c	0.0000±0.0000c	0.0000±0.0000c	0.1160±0.0028b	0.0000±0.0000c	0.0000±0.0000c	0.0002±0.0004c	0.2573±0.0371a
Ethyl pentanoate	1140	0.0004±0.0000c	0.0004±0.0000c	0.0004±0.0000c	0.0146±0.0004b	0.0012±0.0003c	0.0006±0.0001c	0.0005±0.0001c	0.0164±0.0023a
Ethyl hexanoate	899	0.0000±0.0000c	0.0000±0.0000c	0.0000±0.0000c	1.9175±0.0555b	0.0000±0.0000c	0.0000±0.000c	0.0017±0.0001c	3.2533±0.1411a
Ethyl tiglate	1244	0.0007±0.0001c	0.0099±0.0001c	0.0010±0.0001c	0.0047±0.0006a	0.0032±0.0007b	0.0011±0.0002c	0.0014±0.0001c	0.0051±0.0018a
Ethyl 2-hexenoate	1343	0.0000±0.0000c	0.0000±0.0000c	0.0000±0.0000c	0.0309±0.0010b	0.0000±0.0000c	0.0000±0.0000c	0.0000±0.0000c	0.0440±0.0023a
Ethyl caprylate	1439	0.0000±0.0000c	0.0000±0.0000c	0.0000±0.0000c	0.0110±0.0005b	0.0000±0.0000c	0.0000±0.0000c	0.0000±0.0000c	0.0257±0.0010a
Ethyl 3-hydroxyhexanoate	1677	0.0000±0.0000c	0.0000±0.0000c	0.0000±0.0000c	0.0011±0.0027b	0.0000±0.0000c	0.0000±0.0000c	0.0000±0.0000c	0.0015±0.0004a
Acetate esters									

Methyl acetate	848	0.0052±0.0003e	0.0184±0.0012de	0.0624±0.0018d	1.0624±0.0442a	0.0107±0.0011e	0.0236±0.0016de	0.3681±0.0363c	0.7315±0.0478b
Ethyl Acetate	878	0.0071±0.0003c	0.0057±0.0006c	0.0097±0.0005c	2.1079±0.0061a	0.0152±0.0002c	0.0073±0.0010c	0.0228±0.0045c	1.7025±0.0863b
Butyl acetate	1070	0.0000±0.0000b	0.0000±0.0000b	0.0000±0.0000b	0.0526±0.0033a	0.0000±0.0000b	0.0000±0.0000b	0.0000±0.0000b	0.0000±0.0000b
Isopentyl acetate	1136	0.0000±0.0000c	0.0000±0.0000c	0.0017±0.0003c	0.1918±0.0049a	0.0000±0.0000c	0.0000±0.0000c	0.0059±0.0020c	0.1611±0.0781b
2-Heptyl acetate	1380	0.0000±0.0000c	0.0000±0.0000c	0.0000±0.0000c	0.0070±0.0010b	0.0000±0.0000c	0.0000±0.0000c	0.0000±0.0000c	0.0121±0.0031a
Hexyl acetate	1299	0.0004±0.0000e	0.0214±0.0039d	0.0041±0.0004e	0.3365±0.0124a	0.0022±0.0005e	0.0041±0.0008e	0.0309±0.0067c	0.0623±0.1105b
trans-3-Hexenyl acetate	1306	0.0003±0.0001d	0.0032±0.0003cd	0.0016±0.0001d	0.0130±0.0005a	0.0048±0.0006c	0.0018±0.0002d	0.0099±0.0015b	0.0114±0.0041ab
trans-2-Hexenyl acetate	1337	0.0069±0.0001d	0.0415±0.0062c	0.0111±0.0013d	0.0980±0.0020a	0.0040±0.0006d	0.0108±0.0013d	0.0906±0.0183a	0.0621±0.0036b
Other esters									
Methyl butyrate	945	0.0043±0.0003d	0.0047±0.0004d	0.0093±0.0080d	1.3970±0.0060a	0.0000±0.000d	0.0056±0.0012d	0.0634±0.0094c	1.2041±0.0699b
Methyl 2-methylbutyrate	1011	0.0000±0.0000d	0.0000±0.0000d	0.0000±0.0000d	0.0568±0.0015a	0.0000±0.0000d	0.0000±0.0000d	0.0049±0.0005c	0.0537±0.0044b
Methyl hexanoate	1197	0.0000±0.0000c	0.0000±0.0000c	0.0027±0.0003c	3.6695±0.0868a	0.0000±0.0000c	0.0000±0.0000c	0.0594±0.0127c	2.2549±0.1206b
Butyl butylate	1240	0.0000±0.0000b	0.0000±0.0000b	0.0000±0.0000b	0.0331±0.0014a	0.0000±0.0000b	0.0000±0.0000b	0.0000±0.0000b	0.0369±0.0168a
Isoamyl butylate	1263	0.0000±0.0000c	0.0000±0.0000c	0.0000±0.0000c	0.0428±0.0025a	0.0000±0.0000c	0.0000±0.0000c	0.0000±0.0000c	0.0345±0.0060b
Methyl octanoate	1394	0.0000±0.0000c	0.0000±0.0000c	0.0000±0.0000c	0.0392±0.0015a	0.0000±0.0000c	0.0000±0.0000c	0.0001±0.0000c	0.0250±0.0003b
Hexyl butyrate	1417	0.0000±0.0000e	0.0005±0.0005cd	0.0003±0.0000d	0.0035±0.0003a	0.0008±0.0002c	0.0004±0.0001d	0.0005±0.0000bd	0.0025±0.0002b
trans-2-Hexenyl Butyrate	1476	0.0000±0.0000c	0.0000±0.0000c	0.0000±0.0000c	0.0044±0.0003b	0.0000±0.0000c	0.0000±0.0000c	0.0000±0.0000c	0.0065±0.0004a
Benzene and volatile phenols									
Ethylbenzol	1130	0.0034±0.0003d	0.0054±0.0001d	0.0036±0.0004d	0.0172±0.0002b	0.0110±0.0026c	0.0040±0.0010d	0.0062±0.0004d	0.0222±0.0037a
Styrene	1264	0.0004±0.0000c	0.0005±0.0000c	0.0004±0.0000c	0.0109±0.0007a	0.0011±0.0002c	0.0005±0.0001c	0.0012±0.0003c	0.0058±0.0021b
Benzaldehyde	1523	0.0017±0.0001d	0.0018±0.0002d	0.0025±0.0002d	0.2953±0.0301b	0.0025±0.0001d	0.0018±0.0000d	0.0052±0.0007c	0.3861±0.0508a
Clorius	1590	0.0000±0.0000d	0.0000±0.0000d	0.0001±0.0000cd	0.0084±0.0006a	0.0000±0.0000d	0.0000±0.0000d	0.0005±0.0001c	0.0060±0.0005b
Acetophenone	1656	0.0005±0.	0.0005±0.	0.0005±0.	0.0067±0.	0.0004±0.0	0.0004±0.0	0.0004±0.	0.0110±0.

		0000c	0000c	0000c	0001b	001c	000c	0001c	0020a
Ethyl benzoate	1671	0.0000±0.0000b	0.0000±0.0000b	0.0000±0.0000b	0.0107±0.0009a	0.0000±0.0000b	0.0000±0.0000b	0.0000±0.0000b	0.0027±0.0048a
Benzyl acetate	1727	0.0000±0.0000c	0.0000±0.0000c	0.0000±0.0000c	0.0714±0.0051a	0.0000±0.0000c	0.0000±0.0000c	0.0003±0.0000c	0.0166±0.0016b
Naphthalene	1744	0.0037±0.0001d	0.0029±0.0003d	0.0022±0.0002d	0.0182±0.0031a	0.0064±0.0005c	0.0036±0.0005d	0.0025±0.0006d	0.0109±0.0016b
Methyl salicylate	1675	0.0001±0.0000c	0.0003±0.0000c	0.0004±0.0000c	0.0196±0.0016a	0.0028±0.0001b	0.0008±0.0000c	0.0034±0.0007b	0.0208±0.0020a
2-Methylnaphthalene	1839	0.0011±0.0000d	0.0009±0.0001d	0.0007±0.0001d	0.0047±0.0008a	0.0020±0.0002c	0.0011±0.0002d	0.0009±0.0003d	0.0030±0.0004b
Benzyl alcohol	1883	0.0036±0.0000c	0.0013±0.0001c	0.0012±0.0001c	0.0058±0.0018a	0.0036±0.0001b	0.0011±0.0002c	0.0038±0.0007b	0.0036±0.0005b
Phenylethyl Alcohol	1920	0.0001±0.0000c	0.0004±0.0000bc	0.0004±0.0002bc	0.0014±0.0004a	0.0013±0.0001a	0.0005±0.0001b	0.0012±0.0002a	0.0011±0.0001a
p-Cresol	2077	0.0002±0.0000c	0.0002±0.0000c	0.0002±0.0000c	0.0015±0.0006a	0.0010±0.0001b	0.0002±0.0000c	0.0005±0.0002c	0.0019±0.0002ab
o-Cresol	1998	0.0001±0.0000d	0.0002±0.0000d	0.0002±0.0001d	0.0007±0.0001b	0.0011±0.0001a	0.0002±0.0000d	0.0005±0.0001c	0.0005±0.00001c
Benzenol	2012	0.0002±0.0000e	0.0002±0.0000e	0.0002±0.0000e	0.0010±0.0001b	0.0005±0.0001c	0.0002±0.0000e	0.0003±0.0001d	0.0011±0.0001a
Aldehydes and ketone									
Dimethyl diketone	990	0.0038±0.0003c	0.0044±0.0004c	0.0091±0.0014c	1.0595±0.0058a	0.0061±0.0007c	0.0054±0.0005c	0.0579±0.0072b	1.0454±0.0544a
Pentanal	982	0.0090±0.0005de	0.0073±0.0007e	0.0070±0.0002e	0.0725±0.0008a	0.0108±0.0011cd	0.0067±0.0007e	0.0135±0.0015c	0.0673±0.0045b
Ethyl vinyl ketone	1014	0.0238±0.0009b	0.0215±0.0017bc	0.0205±0.0013bc	0.0130±0.0006e	0.0192±0.0024cd	0.0159±0.0025de	0.0218±0.0027bc	0.0306±0.0020a
Isopropenyl ethyl ketone	1069	0.0023±0.0001c	0.0044±0.0004b	0.0048±0.0006b	0.0000±0.0000d	0.0022±0.0002c	0.0028±0.0002c	0.0056±0.0005a	0.0000±0.0000d
Hexanal	1041	0.2323±0.0111c	0.3342±0.0435c	0.2939±0.0326c	0.6557±0.0074b	0.9195±0.1555a	0.2985±0.0484c	0.3972±0.0536c	0.6500±0.2022b
trans-2-Pentenal	1173	0.0090±0.0007e	0.0117±0.0001de	0.0112±0.0004de	0.0127±0.0008cd	0.0160±0.0022b	0.0106±0.0008de	0.0150±0.0039bc	0.0190±0.0002a
3-Hexenal	1146	0.0082±0.0003e	0.0140±0.0017cd	0.0149±0.0012c	0.0117±0.0022cde	0.0375±0.0038a	0.0135±0.0020cd	0.0238±0.0049b	0.0094±0.0033de
Isobutyl ketone	1207	0.0036±0.0001a	0.0036±0.0003a	0.0030±0.0002a	0.0083±0.0001a	0.0073±0.0005a	0.0039±0.0007a	0.0291±0.0434a	0.0090±0.0006a
Heptanal	1187	0.0017±0.0001d	0.0025±0.0004d	0.0022±0.0003d	0.0685±0.0003a	0.0056±0.0010c	0.0023±0.0005d	0.0046±0.0008c	0.0492±0.0020b
trans-2-Hexenal	1329	0.0000±0.0000	0.0000±0.0000	0.0000±0.0000	0.0000±0.0000	0.0000±0.0000	0.0000±0.0000	0.0000±0.0000	0.0000±0.0000
2-Hexenal	1220	0.4320±0.0132d	0.7647±0.0576c	0.6799±0.0494c	0.6708±0.0027c	1.9846±0.2810a	0.6720±0.0837c	1.0277±0.1355b	0.8172±0.0293c

3-Octanone	1281	0.0004±0.0000c	0.0006±0.0001c	0.0006±0.0001c	0.0379±0.0013a	0.0008±0.0001c	0.0005±0.0001c	0.0013±0.0001c	0.0220±0.0017b
Octanal	1280	0.0011±0.0002cd	0.0008±0.0001cd	0.0007±0.0000d	0.0106±0.0007a	0.0013±0.0002c	0.0009±0.0002cd	0.0012±0.0002cd	0.0073±0.0004b
1-Octene-3-one	1438	0.0048±0.0003a	0.0028±0.0046cd	0.0024±0.0005cd	0.0021±0.0000d	0.0040±0.0005ab	0.0032±0.0007bc	0.0039±0.0009ab	0.0032±0.0004bc
cis-2-Heptenal	1323	0.0045±0.0001e	0.0073±0.0008cd	0.0071±0.0008cd	0.0061±0.0004d	0.0083±0.000bc	0.0047±0.0004e	0.0088±0.0014b	0.0108±0.0007a
Sulcatone	1333	0.0014±0.0001f	0.0026±0.0002cd	0.0024±0.0002cde	0.0167±0.0002b	0.0020±0.0001def	0.0016±0.0001ef	0.0033±0.0005c	0.0208±0.0014a
Nonanal	1386	0.0004±0.0000c	0.0004±0.0000c	0.0004±0.0000c	0.0016±0.0003b	0.0017±0.0003b	0.0005±0.0002c	0.0007±0.0002c	0.0060±0.0005a
Sorbaldehyde	1411	0.0041±0.0002c	0.0069±0.0006bc	0.0062±0.0005bc	0.0057±0.0001bc	0.0234±0.0067a	0.0069±0.0015bc	0.0102±0.0011b	0.0070±0.0007bc
trans-2-Octenal	1428	0.0017±0.0001e	0.0026±0.0003cd	0.0027±0.0004cd	0.0079±0.0003b	0.0024±0.0002cde	0.0020±0.0002de	0.0031±0.0006c	0.0164±0.0011a
trans-2-trans-4-Heptadienal	1494	0.0039±0.0003d	0.0061±0.0002d	0.0064±0.0004d	0.0092±0.0004c	0.0141±0.0007a	0.0041±0.0003e	0.0084±0.0012c	0.0126±0.0013b
trans-2-Nonenal	1679	0.0003±0.0000d	0.0005±0.0000cd	0.0005±0.0001cd	0.0063±0.0004a	0.0008±0.0001c	0.0005±0.0001cd	0.0008±0.0002c	0.0052±0.0003b
Cucumber aldehyde	1589	0.0007±0.0000d	0.0011±0.0001d	0.0011±0.0001d	0.0038±0.0003b	0.0020±0.0003c	0.0010±0.0001d	0.0019±0.0004c	0.0046±0.0002a
trans, trans-2,4-Nonadienal	1703	0.0002±0.0000c	0.0003±0.0000bc	0.0003±0.0000bc	0.0030±0.0004a	0.0006±0.0000b	0.0002±0.0000c	0.0004±0.0000bc	0.0031±0.0003a
Isoprenoids									
β-Myrcene	1170	0.0000±0.0000	0.0000±0.0000	0.0000±0.0000	0.0000±0.0000	0.0000±0.0000	0.0000±0.0000	0.0000±0.0000	0.0000±0.0000
D-Limonene	1203	0.0002±0.0000c	0.0002±0.0000c	0.0004±0.0006c	0.0118±0.0014a	0.0000±0.0000c	0.0004±0.0001c	0.0014±0.0003c	0.0096±0.0030b
β-Ocimene	1246	0.0000±0.0000d	0.0000±0.0000d	0.0002±0.0000d	0.0105±0.0003a	0.0000±0.0000d	0.0001±0.0000d	0.0012±0.0002c	0.0115±0.0005a
γ-Terpinene	1255	0.0001±0.0000c	0.0001±0.0000c	0.0002±0.0000c	0.0109±0.0022a	0.0000±0.0000c	0.0002±0.0001c	0.0004±0.0000c	0.0048±0.0002b
Dihydrolinalool	1512	0.0016±0.0002c	0.0013±0.0000c	0.0008±0.0002c	0.0056±0.0011a	0.0001±0.0002c	0.0014±0.0003c	0.0007±0.0002c	0.0025±0.0007b
trans-Linalool oxide	1464	0.0000±0.0000e	0.0003±0.0001d	0.0006±0.0001c	0.0035±0.0002a	0.0024±0.0002d	0.0003±0.0000d	0.0013±0.0001d	0.0022±0.0002a
Morillol	1452	0.0089±0.0003e	0.0231±0.0015b	0.0233±0.0025b	0.0233±0.0002b	0.0176±0.0013c	0.0123±0.0002d	0.0249±0.0025b	0.0303±0.0018a
cis-Linalool oxide	1441	0.0000±0.0000e	0.0007±0.0001d	0.0015±0.0004c	0.0038±0.0003a	0.0004±0.0001de	0.0006±0.0000b	0.0033±0.0004b	0.0032±0.0015b
Linalool	1527	0.0021±0.0001c	0.0050±0.0003c	0.0117±0.0008c	0.9393±0.0590a	0.0082±0.0009c	0.0070±0.0012c	0.0849±0.0095b	0.9278±0.0465a
Levomenthol	1651	0.0008±0.	0.0008±0.	0.0005±0.	0.0037±0.	0.0005±0.0	0.0006±0.0	0.0005±0.	0.0020±0.

		0000c	0001c	0007c	0006a	001c	001c	0001c	0003b
cis-α-Farnesene	1685	0.0000±0.0000c	0.0000±0.0000c	0.0000±0.0000c	0.0069±0.0022b	0.0000±0.0000c	0.0000±0.0000c	0.0000±0.0000c	0.0162±0.0029a
α-Terpineol	1697	0.0013±0.0000c	0.0015±0.0002c	0.0015±0.0003c	0.0414±0.0051a	0.0027±0.0003c	0.0016±0.0003c	0.0036±0.0002c	0.0231±0.0017b
Bisabolene	1792	0.0000±0.0000c	0.0000±0.0000c	0.0000±0.0000c	0.0014±0.0008b	0.0000±0.0000c	0.0000±0.0000c	0.0000±0.0000c	0.0023±0.0004a
Geraniol	1843	0.0000±0.0000c	0.0000±0.0000c	0.0001±0.0000c	0.0036±0.0005a	0.0000±0.0000c	0.0000±0.0000c	0.0002±0.0000c	0.0022±0.0001b
Nerylacetone	1865	0.0003±0.0001a	0.0002±0.0000b	0.0002±0.0000b	0.0000±0.0000c	0.0000±0.0000c	0.0003±0.0000a	0.0003±0.0001a	0.0000±0.0000c
β-Damascenone	1808	0.0023±0.0004bc	0.0037±0.0003ab	0.0036±0.0006ab	0.0003±0.0008ab	0.0004±0.0001c	0.0046±0.0030a	0.0019±0.0002bc	0.0019±0.0001bc
Nerolidol	1961	0.0000±0.0000c	0.0000±0.0000c	0.0000±0.0000c	0.2655±0.03390b	0.0000±0.0000c	0.0000±0.0000c	0.0006±0.0002c	0.2798±0.0233a
Furan									
2-Pentylfurane	1231	0.0013±0.0000b	0.0029±0.0004b	0.0030±0.0003b	0.0341±0.0014a	0.0050±0.0005b	0.0026±0.0005b	0.0023±0.0020b	0.0423±0.0136a
Furfural	1472	0.0013±0.0000a	0.0014±0.0000a	0.0013±0.0002a	0.0007±0.0001b	0.0003±0.0000d	0.0013±0.0002a	0.0002±0.0000d	0.0005±0.0001c
3-Phenylfuran	1880	0.0000±0.0000b	0.0000±0.0000b	0.0000±0.0000b	0.0010±0.0003a	0.0000±0.0000b	0.0000±0.0000b	0.0000±0.0000b	0.0011±0.0002a

Note: WT represents cultivated strawberries 'Sachinoka'. *Mut* represents somaclonal mutant 'Mixue'. RI, retention indices on HP-innowax column. a-f represents the volatile components having significant differences between 'Sachinoka' strawberry and its somaclonal mutant 'Mixue' ($p < 0.05$).

Table S4. The proportion of each major component in the four periods in 'Sachinoka' strawberry (WT) and its somaclonal mutant 'Mixue' (*Mut*)

	WT-Green	WT-White	WT-Turn	WT-Red	Mut-Green	Mut-White	Mut-Turn	Mut-Red
Alcohols	61.41%	54.22%	50.03%	2.22%	31.66%	52.83%	30.24%	2.52%
Acid	0.94%	0.77%	1.72%	38.78%	0.35%	0.60%	3.55%	33.31%
Ethyl esters	0.11%	0.09%	0.20%	17.01%	0.39%	0.23%	0.43%	25.98%
Acetate esters	1.57%	6.87%	7.75%	13.73%	2.18%	4.53%	25.03%	11.75%
Other esters	0.54%	0.43%	1.17%	20.87%	0.05%	0.62%	6.71%	17.14%
Benzene and Volatile phenols	0.80%	0.65%	0.64%	1.00%	1.05%	0.79%	0.75%	1.27%
Aldehydes and Ketone	32.72%	34.59%	35.42%	3.71%	62.64%	37.93%	29.85%	4.64%
Isoprenoids	1.04%	1.44%	1.98%	2.31%	0.86%	1.42%	3.08%	2.94%
Furan	0.87%	0.96%	1.08%	0.38%	0.82%	1.05%	0.36%	0.45%

Table S5. The unique aroma substances in the 'Sachinoka' strawberry

Aroma components	Classification	Period	Relative Content
Isobutyric acid	Acid	Green	0.000208501
Butanoic acid	Acid	Green	0.000142947
Methylethylacetic acid	Acid	Green	0.000135555
Methyl butyrate	other esters	Green	0.004277728
D-Limonene	Isoprenoids	Green	0.000151926
γ-Terpinene	Isoprenoids	Green	7.252E-05
Nerylacetone	Isoprenoids	Green	0.000330989
Butanoic acid	Acid	White	0.000113052
Butyl acetate	acetate esters	Red	0.052619487

Table S6. The unique aroma substances in somaclonal mutant 'Mixue'

Aroma components	Classification	Period	Relative Content
trans-3-Hexenol	Alcohols	Green	0.000599473
Ethyl butyrate	Ethyl esters	Green	0.003761994
Hexyl butyrate	Other esters	Green	0.000782091
cis-Linalol oxide	Isoprenoids	Green	0.000292659
trans-Linalool oxide (furanoid)	Isoprenoids	Green	0.000416069
Ethyl butyrate	Ethyl esters	White	0.001069711
β-Ocimene	Isoprenoids	White	0.000138357
Ethyl hexanoate	Ethyl esters	Turn	0.001659355
Methyl 2-methylbutyrate	Other esters	Turn	0.004850518
Methyl octanoate	Other esters	Turn	0.000120729
Nerolidol	Isoprenoids	Turn	0.000574834

Table S7. Principal component load matrix of volatile components

Volatile compounds	PC1	PC2	PC3	PC4	PC5
Alcohols	0.00957238	0.98585676	0.1501523	0.0666033	0.00954012
Acid	-0.36477647	0.00616727	0.1464225	0.3721668	0.04657792
Ethyl esters	-0.36297505	0.05063711	0.0632333	0.5183155	0.35605858
Acetate esters	-0.36434459	0.03079673	0.1428199	0.4226701	0.09775388
Other esters	-0.36447322	-0.00011642	0.1446656	-0.435025	0.05024322
Benzene and Volatile phenols	0.369264234	0.02657591	-0.0524712	0.2268815	-0.11952753
Aldehydes and ketone	-0.25810688	-0.15284177	0.947808	-0.0970270	-0.03669185
Isoprenoids	-0.36808427	0.01871706	-0.0927242	0.2229273	-0.34112913
Furan	-0.36200364	0.01089189	-0.0316052	0.3315446	0.85257220
